# Assessment of stormwater discharge contamination and toxicity for a cold-climate urban landscape

**DOI:** 10.1186/s12302-022-00619-x

**Published:** 2022-05-13

**Authors:** H. Popick, M. Brinkmann, Kerry McPhedran

**Affiliations:** 1 Department of Civil, Geological, and Environmental Engineering, College of Engineering, University of Saskatchewan, Saskatoon, SK Canada; 2 Global Institute for Water Security, University of Saskatchewan, Saskatoon, SK Canada; 3 School of Environment and Sustainability, University of Saskatchewan, Saskatoon, SK Canada; 4 Toxicology Centre, University of Saskatchewan, Saskatoon, SK Canada; 5 Centre for Hydrology, University of Saskatchewan, Saskatoon, SK Canada; 6 University of Saskatchewan, RM 1A13, Engineering Building, 57 Campus Dr. Saskatoon, Saskatoon, SK S7N 5A9 Canada

**Keywords:** Stormwater, Polyaromatic hydrocarbons (PAHs), Metals, Cold climate, *Raphidocelis subcapitata* (algae), *Vibrio fischeri* (bacteria)

## Abstract

**Background:**

Stormwater is water resulting from precipitation events and snowmelt running off the urban landscape, collecting in storm sewers, and typically being released into receiving water bodies through outfalls with minimal to no treatment. Despite a growing body of evidence observing its deleterious pollution impacts, stormwater management and treatment in cold climates remains limited, partly due to a lack of quality and loading data and modeling parameters. This study examines the quality of stormwater discharging during the summer season in a cold-climate, semi-arid Canadian city (Saskatoon, Saskatchewan).

**Results:**

Seven stormwater outfalls with mixed-land-use urban catchments > 100 km^2^ were sampled for four summer (June–August 2019) storm events and analyzed for a suite of quality parameters, including total suspended solids (TSS), chemical oxygen demand (COD), dissolved organic carbon (DOC), metals, and targeted polyaromatic hydrocarbons (PAHs). In addition, assessment of stormwater toxicity was done using the two toxicity assays *Raphidocelis subcapitata* (algae) and *Vibrio fischeri* (bacteria). Notable single-event, single-outfall contaminant pulses included of arsenic (420 µg/L), cadmium (16.4 µg/L), zinc (924 µg/L), fluorene (4.95 µg/L), benzo[a]pyrene (0.949 µg/L), pyrene (0.934 µg/L), phenanthrene (1.39 µg/L), and anthracene (1.40 µg/L). The IC_50_ in both *R. subcapitata* and *V. fischeri* was observed, if at all, above expected toxicity thresholds for individual contaminant species. Principal component analysis (PCA) showed no clear trends for individual sampling sites or sampling dates. In contrast, parameters were correlated with each other in groups including DOC, COD, TSS, and reduced algal toxicity; and total dissolved solids (TDS), sum of metals, and pH.

**Conclusions:**

In general, stormwater characteristics were similar to those of previous studies, with a bulk of contamination carried by the first volume of runoff, influenced by a combination of rainfall depth, antecedent dry period, land use, and activity within the catchment. Roads, highways, and industrial areas contribute the bulk of estimated contaminant loadings. More intensive sampling strategies are necessary to contextualize stormwater data in the context of contaminant and runoff volume peaks.

**Supplementary Information:**

The online version contains supplementary material available at 10.1186/s12302-022-00619-x.

## Introduction

Stormwater (SW) is water resulting from precipitation events, running off the urban landscape, collecting in storm sewers, and typically being released into receiving water bodies with little to no treatment. Continually increasing urbanization in cities worldwide seals soils, removes vegetation, and changes natural drainage paths. These changes lead to decreasing infiltration capacity of the local landscape and subsequent increases in the volume and flashiness (e.g., quickness of flooding) of SW runoff events [7, 11, 78]. As SW flows over pavements, lawns, driveways, roads, and other urban surfaces, it accumulates high concentrations of contaminants from both point and non-point sources. Thus, relative to natural waters, or even treated municipal sanitary wastewaters (MWW), the quality of SW effluents is generally poor [7, 11, 27, 29, 81].

A wide variety of contaminants are typically distributed across urban surfaces (i.e., non-point sources); however, they may also be present in single origins (i.e., point sources) that contribute to runoff quality. Contamination may include total suspended solids (TSS), total dissolved solids (TDS), nutrients (e.g., nitrogen, phosphorus), pathogens (bacteria, viruses, and coliforms, notably *Escherichia coli*), total and dissolved organic carbon (TOC/DOC), heavy metals, as well as organic chemicals like polyaromatic hydrocarbons (PAHs) [4, 34, 77, 78, 81]. These contaminants can also impact, and be impacted by, other water quality parameters, including pH, electrical conductivity (EC), turbidity (NTU), and chemical or biological oxygen demand (COD/BOD) [10, 34, 38, 39]. For example, the solubility—and consequently aquatic toxicity—of heavy metals is dependent on parameters such as pH, TSS, and organic matter [4, 29, 55, 67, 81]. The complexity of SW matrices makes defining their impacts equally complex since various contaminants may have additive, synergistic, or subtractive effects [24, 25]. Overall, untreated SW is recognized as a significant transporter of municipal contaminants to receiving water bodies [7, 11, 50, 61].

Untreated SW effluents can result in acute or chronic toxicity in receiving water bodies causing physiological changes in aquatic organisms, including impacts on growth, respiration, feeding habits, reproduction, and lethality at elevated concentrations [52, 67]. For example, dissolved PAHs and metals can induce mortality at concentration thresholds observed in SW in *Raphidocelis subcapitata*, *Ceriodaphnia dubia*, and *Daphnia magna* [14, 53]. These toxicity effects are influenced by the presence of TSS, TDS or DOC in solution through the formation of ligands or particle binding. In addition, the bioaccumulation of contaminants can cause chronic toxicity at lower concentrations [7]. Urban stream runoff syndrome, associated with acute mortality in Pacific Northwest coho salmon, has been linked to the quinone derivative of a common rubber tire vulcanizer, 2-anilo-5-[(4-methylpentan-2-yl)amino]cyclohexa-2,5-diene-1,4-dione (6PPD-quinone) [73] and has been observed in geographically separate SWs [17] with implications for local toxicity. Humans may also be exposed to contaminated effluents, especially during flood events, with metals/metalloids such as arsenic, copper, lead, and zinc associated with health effects including cancer, bone disease, hypertension, DNA and enzymatic dysfunction, nervous system damage, infertility, and organ failure [18, 47, 84]. The potentially widespread distribution, coupled with severe impacts, of these SW contaminants necessitate establishing toxicity thresholds as parameters of SW quality which can be included in potential future SW regulations (currently none exist in Canada).

Land use (i.e., industrial, commercial, residential) impacts SW flow quantity and quality when considered in conjunction with climate, atmospheric, and catchment dependent variables. As mentioned previously, urbanized areas provide more contaminated and impervious surfaces leading to rapid discharge peaks and high runoff volumes [7, 68]. For example, industrial land use has been observed to generate more TSS and metals related to roadways and galvanized buildings, while residential and commercial areas generate more TOC and nutrients related to detergents and green space [46]. Green spaces may also be associated with pathogens such as *E. coli*: animal waste-derived fecal coliforms have been observed in SW from undeveloped catchments [7]. Regardless of overall land use, roadways are significant sources of TSS and chemicals contributing to COD from pavement degradation [36]. Vehicular activity further exaggerates road impacts with TSS-bound heavy metals, and PAHs linked to emissions, tire wear, and deterioration [4, 10, 34, 44]. While metals are naturally occurring geogenic elements present in sediments and soils predating industrialization [59], their mobilization in the urban environment is markedly increased by human activities [68, 71]. Overall, SW is considered to be a more prevalent source of metals than other wastewaters [7].

Considering the complex influence of seasonal weather fluctuations and land use on SW quality, its management remains a concern for local governments. An effective management strategy relies on collecting local data to identify the chemical composition and source of contaminants during runoff events. The assessment of the quality and quantity of the City of Saskatoon (CoS) stormwater runoff to the South Saskatchewan River (SSR) in Saskatoon, Saskatchewan, Canada, has been historically limited despite its potential to negatively impact the SSR and downstream municipalities [54, 60, 85]. This study aims to obtain SW quality data for seven major catchments of the CoS by analyzing a suite of pollutants in field samples, including pH, EC, TDS, TSS, COD, DOC, metals, and targeted polyaromatic hydrocarbons (PAHs); furthermore, the toxic potential of filtered SWs will be determined using two toxicity assays *Raphidocelis subcapitata* (algae) and *Vibrio fischeri* (bacteria). These results will be compared to previous literature and regional SW quality data to assess SW quality patterns between or within storm events, or across land uses. Lastly, the impact of land-use categories on catchment-level pollutant loading will be compared to literature-based loading values to assess the viability of grab sampling to estimating seasonal pollutant loadings into the SSR. The inclusion of toxicity assessment, in addition to the typical physicochemical and land-use SW parameters, is not common for SW assessment.

## Methods

### Study area

The City of Saskatoon (CoS) is located in central Saskatchewan and borders both banks of the South Saskatchewan River (SSR). With a total area of 228.1 km^2^ and a population of about 330,000, it is the largest municipality in Saskatchewan, located in western Canada (Statistics Canada 2021). Based on climate data collected by the Saskatchewan Research Council’s Climate Reference Station (CRS), the average daily temperature ranges between approximately 18.7 °C in August and − 14.7 °C in January, with an annual average of 3 °C. The CoS receives an average of 355 mm of precipitation per year, with ~ 50 mm of precipitation falling between the months of November and March as snowfall. The CoS collects rainfall data from eight rain gauges [3] with relevant rain gauges used currently to estimate the individual catchment stormwater volumes and pollutant concentrations. As in many municipalities, the CoS rainfall is often localized making the use of relevant gauges important in the determination of reasonably accurate rainfall volumes.

**Fig. 1:**
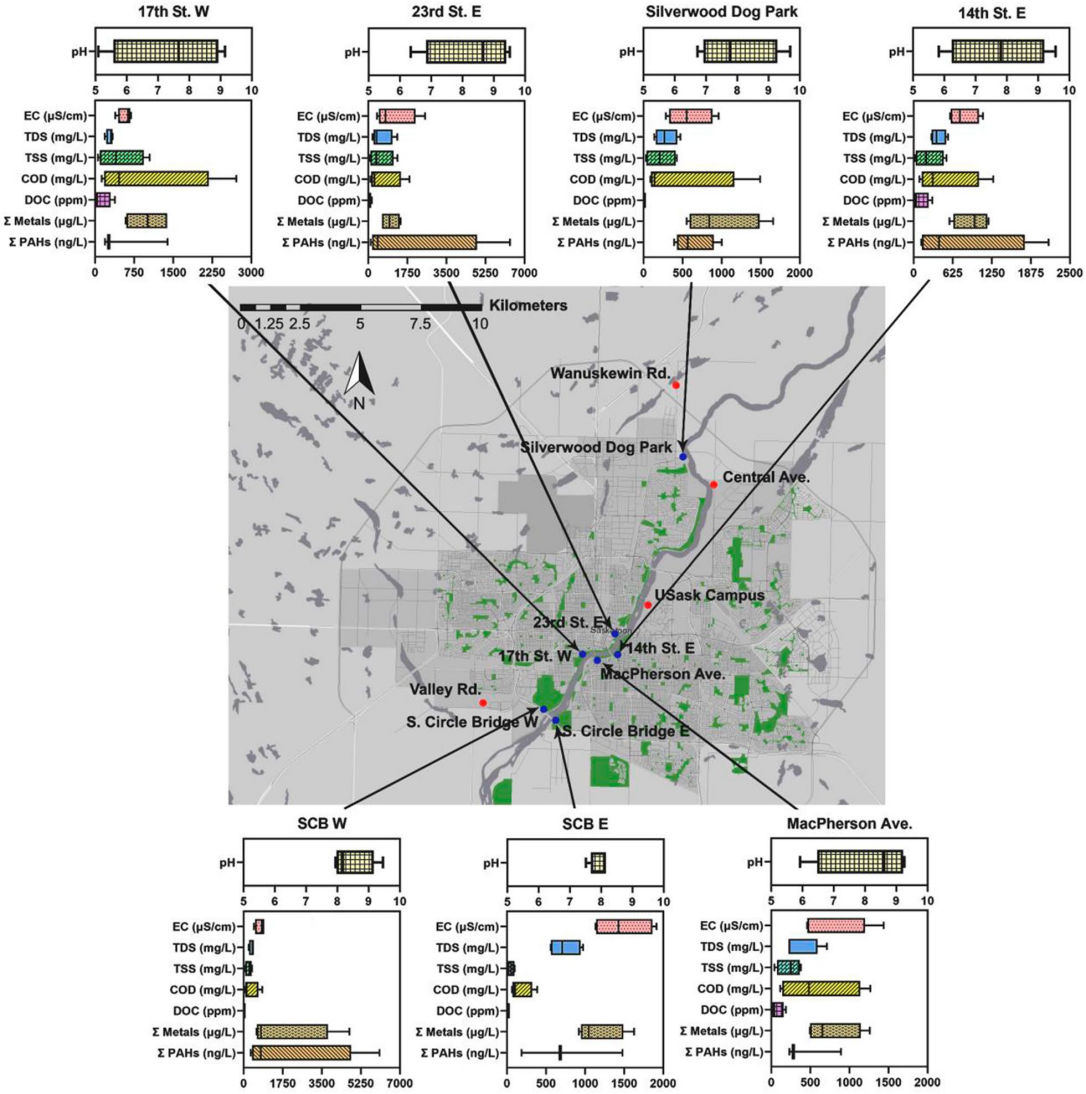
Map of the City of Saskatoon (CoS) and a summary of outfall stormwater (SW) data for the 2019 sampling season. SW outfalls are indicated with blue dots along the river, with red dots indicating snow storage facilities (presented in a parallel study). Box and whisker plots indicate averages for four storm events as summarized in Table 1

Of the CoS storm sewers, seven outfalls with large catchment areas (> 1 km^2^) are considered in the current study (Fig. 1). These seven catchment areas are delineated in the Additional file 1: Figure S1 while sampling outfall locations are presented in Fig. 1. The sampling outfalls chosen for this study (CoS naming conventions in brackets) include those at Circle Bridge on the east side [S. Circle Dr. Bridge East (SCB E)] and west [S. Circle Dr. Bridge West (SCB W)] banks; outfalls close to the CoS central business district (MacPherson Ave.; 14th St. E.; 17th St. W.; 23rd St. E.), and one outfall located in the north end of the city (Silverwood Dog Park). These outfalls are identified with blue markers, while red markers denote snow storage facilities which were sampled as part of a parallel study ([60]; Fig. 1).

### Sampling and laboratory analyses

Grab sampling was used to collect SW from seven outfalls during four wet weather events between June and August 2019. As recorded at the CRS, the first seasonal frost often occurs in September and the last in May; therefore June, July, and August were selected as the time window for ice-free SW. This method of SW sampling has traditionally been the most common due to its simplicity and accessibility [80, 83]; however, drawbacks of this method include the ability only to determine instantaneous pollutant concentrations and the potential for variations in composition occurring prior to laboratory analysis, i.e., during sample transport [83]. Sampling only occurred during storm events if < 3 mm of rainfall was observed 48 h prior to the onset of the storm to allow contaminants to accumulate on land surfaces. Sampling commenced within the first 30 min of the storm (except July 25, where sampling occurred the morning after the storm due to safety concerns of night sampling). Outfalls were approached from above, and a Nalgene sampling pail was lowered into the SW flow close to the mouth of the outfall. Collected samples were then transferred into 4-L and 1-L Nalgene containers. Samples were sealed, labeled, and transported back to the University of Saskatchewan (USask) Environmental Engineering labs, where they were stored at 4 °C prior to analysis.

Several physicochemical, biological, and toxicity parameters were selected to assess the SW quality. The physicochemical analyses included pH, TSS, TDS, EC, COD, TOC/DOC, metals, and PAHs. The biological analyses comprised the enumeration of fecal coliforms, while two standardized laboratory toxicity assays were conducted with *Raphidocelis subcapitata* algae and *Vibrio fischeri* bacteria.

#### Physicochemical analyses

The TSS concentration was measured via vacuum filtration by following Standard Methods 2540 [1], using Whatman™ 934-AH™ glass microfibre filters (1.5 µm). A HACH sensION 156 digital probe was used to measure the pH, TDS, and EC of the samples. To analyze DOC, all samples were extracted through a 0.45-µm Teflon filter using a Luer-Lock 12-mL syringe. Approximately 40 mL of filtered sample was placed in a glass vial and measured with a Lotix combustion TOC analyzer (Teledyne Tekmer, OH, USA) following the manufacturer-provided method. To measure COD, samples were added to VWR Mercury-Free High-Range (20–1500 mg/L) COD digestion vials following the HACH COD Method 8000 [31]. Samples were run in duplicate using either 2 mL of sample or 1 mL of sample and 1 mL of distilled water. Afterward, the COD was measured using a HACH DR/4000U Spectrophotometer (HACH USA, CO, USA) set to 625 nm.

For metals analysis, samples were acidified using 0.02 N nitric acid and vacuum-filtered through a 0.45-µm nitrocellulose filter. A 100-mL sample volume was passed through the filter and the filtrate collected in a Nalgene container. Samples were analyzed using inductively coupled plasma mass spectrometry (ICP-MS) at the USask Department of Geological Sciences or the USask Toxicology Centre. The methods at the Department of Geological Sciences included the use of the PerkinElmer 300D ICP-MS, diluting samples 20 × prior to analysis and using a custom calibration standard (SCP Science) for blanks and standards of 10, 50, and 100 ppb. The certified reference material was NIST-SRM1643f (https://www-s.nist.gov/srmors/msds/1640A-MSDS.pdf). At the Toxicology Centre, samples were analyzed using an Agilent 8800 ICP-MS QQQ Triple Quadrupole mass spectrometer (Agilent, Santa Clara, USA). OmniTrace Ultra nitric acid (HNO_3_) (w/w) (Millipore-Sigma, Ontario, Canada) was used for blanks, standards, and sample solutions. High-purity standard stock solutions (1000 mg/L) were purchased from Delta Scientific (Mississauga, Canada). The calibration standard solution containing 22 multi-elements was supplied by SCP Science (Quebec, Canada). The standard reference material, natural water 1640a, was supplied by the National Institute of Standards and Technology (NIST) (Gaithersburg, USA). Limits of detection and quantification for each method are included in Additional file 1: Table S1.

Samples for PAH analyses were pre-filtered (Whatman™ GF/F glass microfibre filters (0.7 µm) to remove high TSS concentrations prior to solid-phase extraction (SPE) to eliminate clogging of the filter. Subsequently, samples were analyzed following the method outlined in Everitt et al. [22]. Briefly, samples were spiked with deuterium-labeled internal standard mix (500 mg/L of acenaphthene-d_10_, chrysene-d_12_, and phenanthrene-d_10_ in acetone) provided by Sigma Aldrich (Oakville, ON) and extracted using solid-phase extraction (SPE; Waters Oasis HLB). Extracts were analyzed for PAH concentrations using gas chromatography–mass spectrometry (GC–MS). A Thermo Trace 1300 gas chromatograph with a Thermo ISQ 7000 quadrupole mass detector was used for analysis. Helium (99.999% purity) was used as the carrier gas to separate the PAHs using an Agilent DB-5 ms (60 m × 250 μm I.D., film thickness 0.1 μm) fused silica capillary column. Limits of detection and quantification for each method are included in Additional file 1: Table S1.

#### Biological analysis

Coliforms were enumerated using two different media: m-ColiBlue24 Broth PourRite Ampules (HACH USA, CO, USA) added to Fisher-Scientific Petri dishes and 3 M Petrifilm *E. coli* film plates. Samples added to Petri dishes were first processed via a membrane filtration method [32]. Petrifilm samples were prepared by adding 1 mL of unfiltered sample directly to the Petrifilm according to manufacturer-provided methods [2]. All samples were incubated at 37.5 °C for 24 h before colony-forming units (CFUs) were enumerated.

#### Toxicity analyses

The first toxicity assay was inhibition of the luminescence of *V. fischeri* aquatic bacteria. The method used is described fully in EPS 1/RM/24 (Environment [21] using a Microtox M500 instrument (Modern Water, DE, USA). Briefly, the Osmotic Adjustment Solution (OAS) required to ensure freshwater samples possess appropriate salinity for the marine bacteria was used. Phenol (60 mg/L) was used as a positive control and 20% sucrose as the diluent (SD) as recommended in the protocol for freshwater samples for increased test sensitivity to metals. The SD was also used as the negative control. This method was adapted to measure the luminescence inhibition of dilution series in 96-well microplates using an OptimaSTAR plate analyzer. Samples were pre-filtered [Whatman™ GF/F glass microfibre filters (0.7 µm)] before testing. Prior to the test, 10 mL each of the samples, OAS and SD were refrigerated for one hour before being transferred to 25-mL beakers. Plates were run both inoculated and uninoculated to correct for background luminescence. To inoculate the wells, 1 mL of Microtox Reconstitution Solution was mixed with a vial of lyophilized *V. fischeri* strain NRRL B-11177 (Modern Water, USA); 0.3 mL of this solution was further diluted with 3 mL of SD. This solution was placed in a multichannel pipette reservoir on ice. Placing the plates on a paper towel over ice and using a multi-pipettor, plates were inoculated with 8 µL of the bacteria solution. Luminescence readings were taken immediately, after 5 min, and after 15 min, with plates remaining on ice between measurements. Due to the configuration of the plates, samples and standard were run in triplicate as seven-step dilutions while the phenol was a six-step dilution series. Values for both samples and phenol are reported as the IC_50_ and IC_10_ relative to the negative control at 15 min. The IC_x_ is defined as the concentration of the bulk SW sample required to inhibit luminescence expression by 10% or 50% relative to the negative control.

The second bioassay included 72-h chronic toxicity of SW to *R. subcapitata* green algae following EPA Method 1003.0 [23]. Prior to analysis, a dilution series of inoculated algae stock was used to establish a linear relationship between fluorescence and cell count. Samples [pre-filtered using Whatman™ GF/F glass microfibre filters (0.7 µm)] were prepared in 1-mL wells of 24-well microplates in a configuration of four replicates of a stock negative control and a five-step dilution series. Wells were inoculated with 100 µL of inoculum (1,000,000 cells/mL) as prepared according to the protocol to meet appropriate cell densities (10,000 cells/mL in wells at the start of the test). Using fluorescence as a proxy for algae cell count, growth inhibition was measured at an emission wavelength of 535 nm as a function of fluorescence at 0 h and at 72 h. Cell counts were verified directly from one random control well per plate at 0 h to ensure proper inoculation and again at 72 h to verify fluorescence emission corresponded with cell population. Fluorescence and cell count were measured using the Tecan Spark^®^ multifunction plate reader (Tecan Trading AG, Switzerland). Sample concentration in wells ranged from 100 to 6.25%, both inoculated and uninoculated to account for background fluorescence. Initially, the IC_50_ at 72 h was calculated; as the IC_50_ was not observed at full concentration for all samples, the IC_10_ was additionally calculated (see “Statistical analysis” section). The IC_x_ is defined similarly to the *V. fischeri* bioassay but in relation to expression of fluorescence (as a proxy for cell count) instead of luminescence.

### Rainfall, runoff coefficients, and site mean concentration (SMC) values

Breakdowns of individual land-use areas within each of the seven CoS catchments have been previously determined [3] and are included currently in Additional file 1: Figure S2. Briefly, to determine SW outfall loadings (*L*) into the SSR values needed to be determined including the individual catchment runoff coefficients (CR), the precipitation (*P*), and the site mean concentration (SMC).

The land-use classifications considered herein are presented in Additional file 1: Table S2 including eight classes of single-family residential (SR), multi-family residential (MR), roads (R), highways (HW), commercial (CM), industrial (IN), green (GR), and agricultural (AG). Each of these classifications has been designated a runoff coefficient value by the CoS. The weighted overall CR values for each catchment (mixed land use) were calculated using Additional file 1: Figure S2 and Additional file 1: Table S2 information for use in the loading calculations. The precipitation data for the CoS were taken from rain gauge data available as discussed in “Study area” section.

A given rainfall event’s event mean concentration (EMC), obtained by dividing the total pollutant mass by the total event volume to obtain flow-weighted average concentrations, can be used to develop site mean concentrations (SMC). SMC values constitute the geometric mean of multiple rainfall events’ EMC over a time interval [92] and are considered the most accurate measure of the average pollutant concentrations as it is measured as event-volume-weighted mean values of EMCs [39]. There is no existing SMC data for the current study catchment areas so SMC values were considered based on averages of land use classifications found in previous studies, including Melanen [86], Mitchell [93], Nordeidet et al. [87], and Järveläinen et al. [39] (Additional file 1: Table S3).

Following the modeling methods of Järveläinen et al. [39], Eqs. (1) and (2) were used to calculate monthly pollutant loads:

1 $${L}_{\mathrm{ua}}=CR\cdot P\cdot SMC,$$

where *L*_ua_ (kg/km^2^) is the monthly unit area load, CR (dimensionless) is the runoff coefficient (outlined in Table 1), *P* (mm) is the monthly precipitation depth, and SMC (mg/L) is the characteristic event-volume weight SMC:

2 $${L}_{\mathrm{tot}}= {L}_{\mathrm{ua}}\cdot A,$$

where *L*_tot_ (kg) is the monthly pollutant export rate, and *A* (km^2^) is the total area occupied by the individual land use class.

**Table 1:** Overview of analyzed stormwater quality parameters for the 2019 sampling season

Date	Sites	pH	TDS	EC	DOC	COD	TSS
		(–)	(mg/L)	(μS/cm)	(mg/L)	(mg/L)	(mg/L)
June 12, 2019	7	8.12 (0.90)	648 (378)	1296 (733)	36.2 (22.6)	289 (229)	129 (105)
June 20, 2019	7	9.18 (0.52)	375 (114)	768 (228)	12.8 (4.4)	246 (168)	451 (315)
July 25, 2019	7	8.19 (0.10)	326 (145)	667 (290)	12.8 (4.1)	93.1 (27.4)	44.1 (22.8)^b^
August 22, 2019	7	6.48 (1.00)^a^	346 (235)	707 (468)	154 (145)^a^	1401 (743)^a^	494 (386)
Average (SD)	7	7.99 (1.12)	424 (151)	859 (294)	53.9 (67.5)	508 (602)	280 (226)

Parameters are grouped by sampling event (date) with average (standard deviation) across seven sites presented for each sampling event. Additional file 1: Table S2 includes individual outfall information for each of these sampling events

^a^Significantly different (*p* ≤ 0.05) from average of other storm events

^b^Significantly different from June 20 to August 22 events

### Statistical analysis

To prepare the algae and Microtox data for statistical analysis, the background fluorescence or luminescence of uninoculated samples was subtracted from that of inoculated samples to remove any baseline fluorescence/luminescence. The exponential growth rate between the initial and endpoints was calculated and normalized as a percent of the average growth observed in the negative control for both procedures. With a normalized growth and luminescence inhibition determined for every dilution series, the datasets could be transferred to GraphPad Prism 9 (GraphPad Software, San Diego, CA) for statistical analysis. Within GraphPad Prism, four-parameter logistic regression was used to fit curves to the growth rate inhibition data to obtain the 72-h (for algae) or 5- and 15-min (for Microtox) IC_50_ values and related 95% confidence intervals (CI) for each analyzed sample. As the IC_50_ was not observed for many algae samples, the IC_10_ and its 95% CI was also interpolated from the curve. These results were then used for one-way analysis of variance (ANOVA) with Tukey’s ad hoc tests, correlation, and *t* test analyses.

Two principal component analysis (PCA) scenarios were considered using GraphPad Prism 9 for comparison between sampling sites/dates using variables including pH, TDS, TOC/DOC, COD, fecal coliforms, TSS, sum of metals, sum of PAHs, 1/EC_10_ and 1/EC_50_ for algae and Microtox, respectively (Table 2; Additional file 1: Tables S5, S7, and S8). Following this initial PCA, a second analysis was done where the toxicity variables were excluded due to their limited data availability as compared to the more extensive data available for the other variables. This second analysis included data from Additional file 1: Tables S5, S7, and S8.

**Table 2:** The 72-h growth inhibition IC_50_ and IC_10_ for algae (*R. subcapitata*) and 5- and 15-min IC_50_ for Microtox (*V. fischeri*)

Sampling date	Sampling site name	Algae		Microtox	
		EC_50_ (CI 95%)	EC_10_ (CI 95%)	IC_50-5 min_ (CI 95%)	IC_50-15 min_ (CI 95%)
June 12	SCB E	92.1 (79.9–114)	30.5 (22.0–41.2)	–	–
	MacPherson Ave	ND	100 (NC)	49.6 (15.6– > 100)	60.8 (18.9– > 100)
	17th St. W.	81.9 (73.8–92.1)	31.6 (24.9–40.0)	ND	52.6 (NC)
	23rd St. E.	101 (92.5–114)	47.9 (37.9–59.3)	NC	52.3 (NC)
June 20	23rd St. E.	ND	97.7 (33.8–100)	ND	57.6 (NC)
	Silverwood Dog Park	–	–	ND	58.6 (45.0–71.2)
July 25	SCB E	–	–	28.5 (16.3–54.9)	44.8 (28.2–72.3)
	MacPherson Ave	ND	76.4 (56.1–88.6)	ND	ND
	14th St. E.	–	–	79.2 (59.5–98.7)	52.0 (NC)
	23rd St. E.	ND	90.7 (26.0–100)	ND	71.3 (NC)
	Silverwood Dog Park	109(98.3–127)	35.3 (23.6–44.0)	ND	52.7 (NC)
August 22	SCB W	ND	85.9 (60.8–96.2)	ND	ND (33.92–NC)
	MacPherson Ave	95.0 (68.7–180)	16.6 (8.1–30.5)	62.0 (28.5–ND)	60.7 (42.5–NC)
	14th St. E.	ND	ND	49.6 (15.6–ND)	60.8 (18.9–ND)
	17th St. W.	ND	24.4 (13.1–37.3)	88.2 (69.0–143)	53.9 (NC–59.6)
	23rd St. E.	ND	ND	ND	29.6 (19.9–45.3)
	Silverwood Dog Park	ND	ND	ND	52.8 (NC)

Results are presented as percent of total sample concentration required to read the endpoint. IC_50_ and IC_10_ values were generated using GraphPad Prism as a dose–response curve (see “Methods” section) of, in the case of *R. subcapitata*, the initial (*t* = 0) fluorescence observed after 72 h, and in the case of *V. fischeri*, normalized luminescence as a percent of the negative control

*ND* no observable toxicity detected at the respective threshold; *NC* not calculated; *–* sample not tested

## Results and discussion

The Results and discussion will be considered in three sections including: (1) “Physicochemical data”; (2) “Toxicity assessment”; and (3) “Land use management”. The physicochemical analyses section will include a discussion of results in five subsections, with one covering the pH, TDS, and EC; a second for DOC, COD, and TSS; a third for *E. coli*; a fourth including metals and PAHs; and a fifth for PCA excluding toxicity data. The toxicity section presents the two toxicity assays *R. subcapitata* and *V. fischeri*, along with a PCA section including toxicity data. The final land-use section will be used to discuss the impacts of land-use on the individual catchment area estimated pollutant loadings into the SSR. Figure 1 presents a map of the CoS and a summary of all physicochemical, metals, and PAH data in the form of box-and-whisker plots. Further details will be discussed in each of the following subsections.

### Physicochemical data

A summary of all physicochemical data grouped by the four sampling dates (with seven sites each) is presented in Table 1 with a summary of all data collected shown in box-and-whisker plots in Fig. 1. In addition, a summary of data grouped by sampling site (Additional file 1: Table S4) and outfall-specific data grouped by sampling date (Additional file 1: Table S5) are presented in the Additional file 1.

#### pH, total dissolved solids (TDS), and electrical conductivity (EC)

The event-average pH, TDS, and EC values for all samples collected in 2019 were 7.99, 424 mg/L, and 859 µS/cm, respectively (Table 1). Event-average pH values were highest on the June 20 sampling date with an average of pH 9.18 (Additional file 1: Table S5). Significantly lower pH values relative to all events occurred on August 22 (average pH 6.48) (*p* ≤ 0.05). No significant difference was found relative to the July 25 or June 12 samples, likely due to the pH range observed on these dates (pH 7.02–9.52). Large pH ranges were also observed on August 22 despite the lower overall pH values, with a low pH of 5.09 for 17th St. W. and a high pH of 7.94 for SCB W (Additional file 1: Table S5). These values were both markedly lower than the average values for each of these sites of pH 7.39 and 8.44, respectively. Lower pH values for August 22 samples are likely related to road tar application performed by the CoS in the days immediately before the storm event. Overall, the pH variability appears to be more closely associated with the sampling date than the specific outfall being sampled; however, there are still differences between the individual outfalls, which will be more closely considered in the Land-use management “Land use management” section.

The TDS and EC are typically associated parameters being determined from the same meter; therefore, they will be discussed together in this section. The range of TDS over the entire year is comparable to that observed in other cold climates [72]. The June 12 sampling date produced the highest average values for both parameters with 648 mg/L and 1296 µS/cm, respectively, but there was no significant difference in the dataset relative to other events (*p* > 0.05). However, the elevated concentrations on June 12 were largely due to two sites in particular, including SCB E (974 mg/L and 1914 µS/cm) and 23rd St. E. (1295 mg/L and 2550 µS/cm), respectively (Additional file 1: Table S5). Unlike the pH, the EC and TDS concentrations were elevated at these two sites in general for all sampling dates (Additional file 1: Table S4). Low-volume flow has been observed consistently at these outfalls during dry-weather sampling and surveying, and pipe corrosion may contribute to these differences: Borris et al. [12] found SWs eluted zinc from galvanized pipes and constant dry-weather flows may corrode certain outfalls relative to those observing no dry-weather flow. Nearby traffic surfaces also potentially contribute TDS at minimal runoff volumes (see “Land use management” section).

The two storm events with the greatest TSS, June 20 and August 22, also possessed the highest and lowest average pH values, respectively (Additional file 1: Table S5). The high concentration of TSS relative to other contaminant species (notably EC and TDS, which are comparable in June 20 and August 22 samples to the low-TSS July 25 event) and its event-specific origins may explain the difference. High pH in spring or early summer events is likely contributed by spring weathering of traffic surfaces, as high pH and alkalinity have been observed alongside high TSS in roadside snowbanks [56]. Furthermore, concrete surfaces—regardless of use—contribute eroded particles to alkaline SWs. Previous studies found SW pH increased from 7.0–7.3 to 8.1–9.3 in all test SWs when conveyed in a concrete sewage pipe [12] and from 7.4 to 7.8–8.6 after infiltrating a porous cement pavement [41]. Thus, the high-intensity June 20 storm likely contained eroded concrete particles at the time of sampling. This could explain why average June 20 pH was elevated across all samples relative to other events (Table 1), as high-rainfall-intensity TSS erosion would have been captured during the sampling event (while the June 12 event was low-intensity and the peak of the July 24 event was missed).

**Fig. 2:**
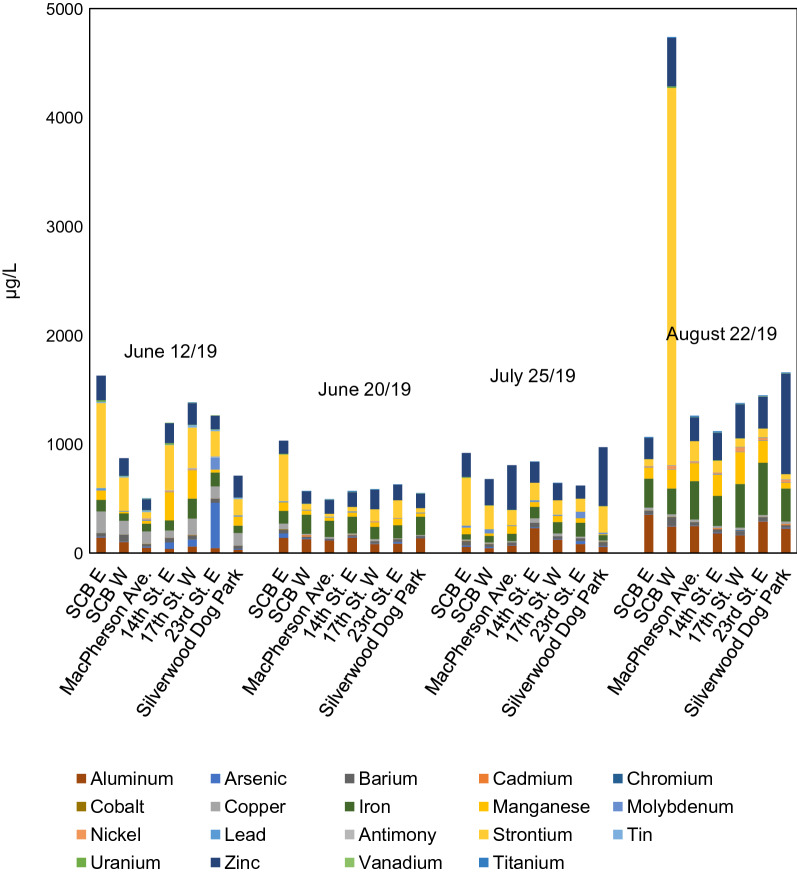
Average measured stormwater dissolved metals concentrations for four sampling dates and seven catchment areas in 2019. Samples were pre-filtered with nitric acid prior to analysis (see “Methods” section). All sampling data values can be found in Additional file 1: Table S7

Relatedly, it is assumed uptake of road tar and its associated metals and chlorinated organic compounds contributed to SW acidification during the August 22 event: low pH values in August samples are accompanied by two-fold greater concentrations of dissolved aluminum and iron relative to all other sampled events [see Fig. 2 and “Heavy metals and polyaromatic hydrocarbons (PAHs)” section], which have been previously associated with higher concentrations of chloride and lower pH values in SWs [6, 62]. Though chloride testing of the current SW samples was limited, the August 22 SCB E sample contained more chloride than all June 12 and July 25 samples tested (Additional file 1: Table S6). In cold climates, TDS and EC are a major toxicity concern due to the practice of road salting in the winter months. With the exception of the August 22 samples, the summer chloride concentrations in this study falls into the same range as previous observations of summer SW across cold and temperature climates [49, 51]. As no early spring storm events occurred over the study period due to an extensive period without rainfall, the potential for spring SW to exhibit elevated TDS and EC relative to summer SW remains to be examined.

#### Dissolved organic carbon (DOC), chemical oxygen demand (COD), and total suspended solids (TSS)

The event-average DOC, COD, and TSS were 53.9 mg/L, 508 mg/L, and 280 mg/L, respectively (Table 1). Each of these parameters had maximum average values on August 22 with 154 mg/L, 1,401 mg/L, and 494 mg/L, respectively, and minimum average values on July 25 with 12.8 mg/L, 93.1 mg/L, and 44.1 mg/L, respectively (Additional file 1: Table S5). The DOC and COD were significantly higher on August 22 relative to other events, while the TSS were significantly higher on June 20 and August 22 relative to July 25, but not June 12 (*p* ≤ 0.05) (Additional file 1: Table S5). Despite the high concentrations on August 22 overall, the individual sites had wide ranges in concentrations, including 12.1–378 mg/L for DOC, 388–1847 mg/L for COD, and 97–1304 mg/L for TSS (Additional file 1: Table S4). Relative to the August event, the ranges for DOC, COD, and TSS on the other three sampling days were 3.14–13.3%, 5.04–45.3%, and 5.76–79.7% smaller, respectively. In addition, the site-specific data indicate a clear trend with the MacPherson Ave., 14th St. E., 17th St. W., and 23rd St. E. having elevated concentrations for these parameters versus the lower concentrations for SCB E, SCB W, and Silverwood Dog Park (Additional file 1: Table S5). Clearly, these parameters vary in both temporal (sampling date) and spatial (site) metrics.

While BOD and COD are commonly used to measure waste-related oxygen demand, measuring organic carbon content is increasingly preferred as techniques for extracting and measuring TOC and DOC become more accessible. As DOC values for the June 20 and July 25 events are comparable, the contaminant peak of these and the June 12 event may have been missed (Table 1). Samples from outfalls located near the geographic center of the CoS displayed a fivefold increase in DOC concentration on June 12 as compared to June 20; these same outfalls possessed the upper range of DOC concentrations observed on August 22 (Additional file 1: Table S5). Relatively larger proportions of residential and commercial land use in city-center catchments likely contribute to elevated DOC and COD. The June and July samples possess COD ranges comparable to other SWs: Lee et al. [42] found a COD range of 10–360 mg/L per storm event, and Zhang et al. [82] observed COD event concentration peaks between 400 and 750 mg/L. Lee and Bang [43] found COD ranges of 13–2796 mg/L for residential areas and 10–810 mg/L for industrial areas, similar to August samples in this study. As previously discussed with respect to pH, road tarring occurred the day before the August 22 event with significant quality impact: organic contaminants related to tar particles are reflected by overall higher concentrations for DOC, COD, and TSS and more acidic pH for this sampling event [82].

The relatively low TSS of the July 25 event might be due to sampling after the bulk of the runoff had already entered the SSR. Across the CoS, the June 12 event had low rainfall depth relative to the June 20 event. The lack of intensity or runoff volume likely explains the difference in average TSS between the two events. Milukaite et al. [55] found an average TSS of 920 mg/L with a range of 48–3640 mg/L, comparable to ranges of 8.3–2796 mg/L found in separated sewers by Lee and Bang [43] and 34–2288 mg/L found in cold-climate highway runoff by Mayer et al. [51]. COD values of 14–320 mg/L Zgheib et al. [81] and 70–1455 mg/L [43], which are ranges comparable to those observed in this study. Somewhat lower TSS (66–937 mg/L) and COD (63–146 mg/L) ranges were compiled across multiple sources of literature by Göbel et al. [29],however, these datasets were published between 1975 and 2001, and SW quality has since changed significantly [76].

#### Coliform analysis

The highest average coliform values were for the August 22 sampling at 4625 *E. coli*/100 mL, followed closely by the July 25 value of 4688 *E. coli*/100 mL (Additional file 1: Table S5). The remaining two sampling dates were lower at 794 and 272 *E. coli*/100 mL for June 20 and June 12, respectively, though no significant difference was observed (*p* > 0.05). On a site basis, there were four sites with averages exceeding 2000 *E. coli*/100 mL, including SCB W (2283 *E. coli*/100 mL), MacPherson Ave. (5963 *E. coli*/100 mL), 14th St. E. (4070 *E. coli*/100 mL), and 23rd St. E. (5000 C *E. coli*/100 mL) (Additional file 1: Table S4). In contrast, the remaining three sites, including SCB E, 17th St. W., and Silverwood Dog Park, had averages below 400 *E. coli*/100 mL. Coliform concentrations varied widely when present, for example, the MacPherson Ave. and 23rd St. E. sampling sites had ranges of < 1–> 9999 *E. coli*/100 mL over the four sampling dates. Overall, no clear trends could be determined based on the current results.

The ranges of enumerable coliforms observed in this study are comparable to a previously reported range of 388 to > 16,000 *E. coli*/100 mL [33]. Selvakumar and Borst [69] reported a range of 1500–8500 *E. coli*/100 mL, where land use did not significantly influence *E. coli* concentrations, but coliform concentration increased with impervious surface density, an observation they corroborated with previous literature. Such high variability between events was similarly observed by McCarthy et al. (2011), who found neither antecedent dry period nor storm intensity explained this variability. Nevertheless, the highest coliform counts in this study were driven by the SCB W, MacPherson Ave., 14th St. E., and 23rd St. E. catchments, though guideline-exceeding counts were found in all samples. Health Canada’s Guidelines for Canadian Recreational Water Quality [89] are ≤ 200 *E. coli*/100 mL for the geometric mean concentration of five samples and ≤ 400 *E. coli*/100 mL for a single sample maximum concentration. The Saskatchewan Water Security Agency recommends in their Sewage Works Design Standard [90] an objective of ≤ 200 *E.* coli/100 mL fecal coliform concentrations in treated wastewater effluent. Dry-weather outfall sampling in summer 2018 has previously reflected relatively high coliforms at the 23rd St. E. outfall, but not at the 14th St. E. outfall; there may be persistent or emerging coliform sources along the reach to the outlet. Coliform/*E. coli* results generally appear to be uninformative and may be of minor value for determination in future SW studies.

#### Heavy metals and polyaromatic hydrocarbons (PAHs)

A total of 18 metals were detected in SW samples as summarized in Fig. 2 with all sampling data for individual dates and outfalls shown in Additional file 1: Table S7. Generally, aluminum, copper, iron, manganese, strontium, and zinc were present in all samples. Dissolved arsenic, copper, and zinc results regularly exceeded Canadian Council of Ministers of the Environment (CCME) guidelines [91] as shown in Additional file 1: Table S7, with threshold-exceeding spikes observed for aluminum, cadmium, iron, and lead. It must be noted ionic species related to hardness were not analyzed as part of the study and the conservative guidelines for unknown hardness were used. The bioavailability of these components may be better expressed using biotic-ligand-model normalized values. Copper was generally an order of magnitude higher in June 12 samples relative to later-season samples; conversely, zinc concentrations were elevated in July and August relative to June. Notable contamination events include discharges of strontium from the SCB E outfall on June 12 (782 µg/L) and the SCB W outfall on August 22 (3463 µg/L); a discharge of arsenic from the 23rd St. E. outfall on June 12 (420 µg/L); and two discharges of zinc from the Silverwood Dog Park catchment on July 25 and August 22 (541 µg/L and 924 µg/L, respectively).

The trends in aluminum and iron may reflect first flush behavior for these parameters, though August 22 concentrations may be elevated due to erosion (as rainfall was brief and intense) or traffic-based deposition from the long antecedent period. Arsenic and strontium spikes in June 12 samples may derive from spring-weathered preserved wood (arsenic) [37] or flares, greases, glass products, and fresh concrete (strontium) [57]. The two spikes of zinc from the Silverwood Dog Park catchment imply potential point-source releases from industrial land use. Seasonal trends in copper and zinc concentrations have been previously observed [48]; additionally, zinc and copper have commonly been found to have the highest concentration of all metals in SW [19, 27, 30, 68, 81]. Elevated zinc levels have been connected with galvanized SW sewer pipes [12]. This was generally consistent with the data, though aluminum and iron were often more abundant, potentially as a reflection of local geogenic concentrations [59]. Mayer et al. [51], Galfi et al. [26], Sakson et al. [68], and Zgheib et al. [81] report similar ranges across cadmium, chromium, copper, iron, and zinc, but the latter two found lead concentrations an order of magnitude greater, compared to this study.

**Fig. 3:**
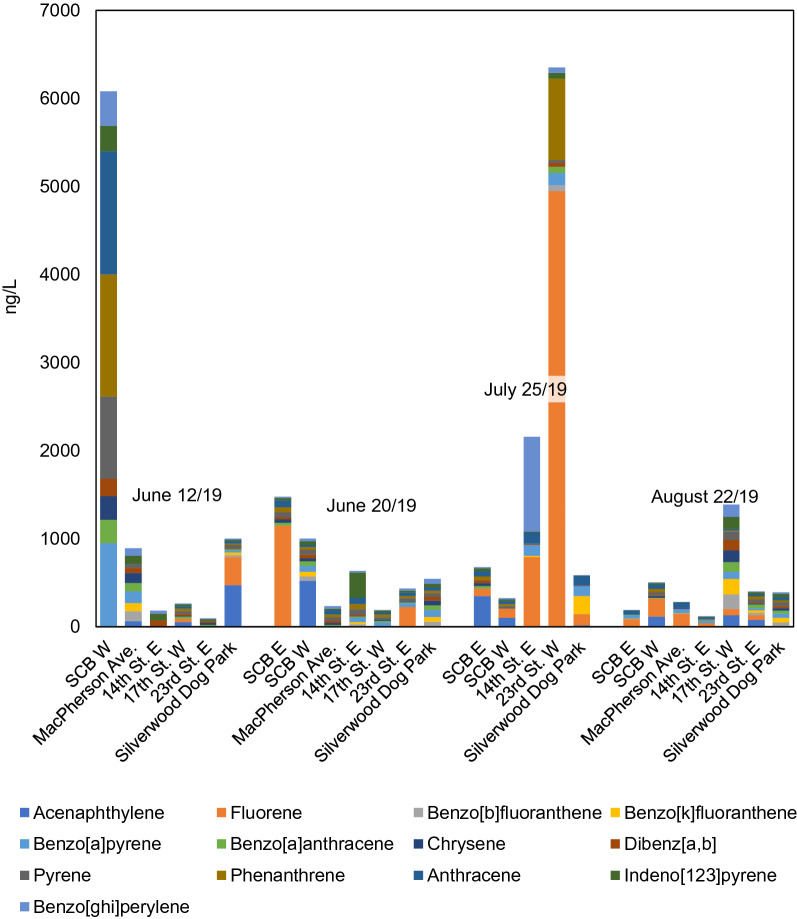
Average stormwater filtered PAH (< 0.7 µm) concentrations for four sampling dates and seven catchment areas in 2019. All sampling data values can be found in Additional file 1: Table S8

A total of 13 PAHs were identified at detectable concentrations as summarized in Fig. 3 with all sampling data for individual dates and outfalls shown in Additional file 1: Table S8. The ∑PAHs was generally below 1 µg/L in the dissolved fraction of SW samples. Fluorene was abundant in the SCB E June 12 sample, the 14th St. E. July 25 sample, and most notably, the 23rd St. E. July 25. Concentrations of anthracene, pyrene, and benzo[*a*]pyrene exceeded the CCME guidelines (Additional file 1: Table S8). Fewer samples exceeded the threshold for benz[*a*]anthracene, and only the SCB W June 12 and 23rd St. E. July 25 samples contained threshold-exceeding amounts of fluorene and phenanthrene.

PAHs in this study were comparable to combined dissolved and particulate findings by Zgheib et al. [81]. Mayer et al. [51] observed that aqueous PAHs were below instrument detection limits and that 91% of PAHs were particulate-bound. Particle-bound PAHs tend to have high molecular weight [4, 27, 49, 64, 81], while low-molecular-weight PAHs predominantly are found in dissolved phases [27]. This would explain some of the unremarkable PAH concentrations found in filtered SW samples, especially high-TSS August 22 samples, as the bulk of PAHs would remain within the filter sediment. The hydrophilic nature of LMW PAHs may also explain the elevated PAHs in July 25 samples relative to August 22: the high presence of benzo[*ghi*]perylene, fluorene, and phenanthrene present in the July 25 samples may have originated from roadway degradation [4] or deposition from emissions [65]; these high concentrations were not observed in the August 22 samples despite road tarring activities. Fluorene and phenanthrene have been previously measured among the most abundant PAHs in road runoff [16]. However, this is speculation, and the presence of dissolved PAHs in July 25 samples may be attributed to other factors, such as the low overall concentration in July 25 samples encouraging partitioning of LMW PAHs from road-wear-related particles in runoff.

#### Principal component analysis excluding toxicity data

**Fig. 4:**
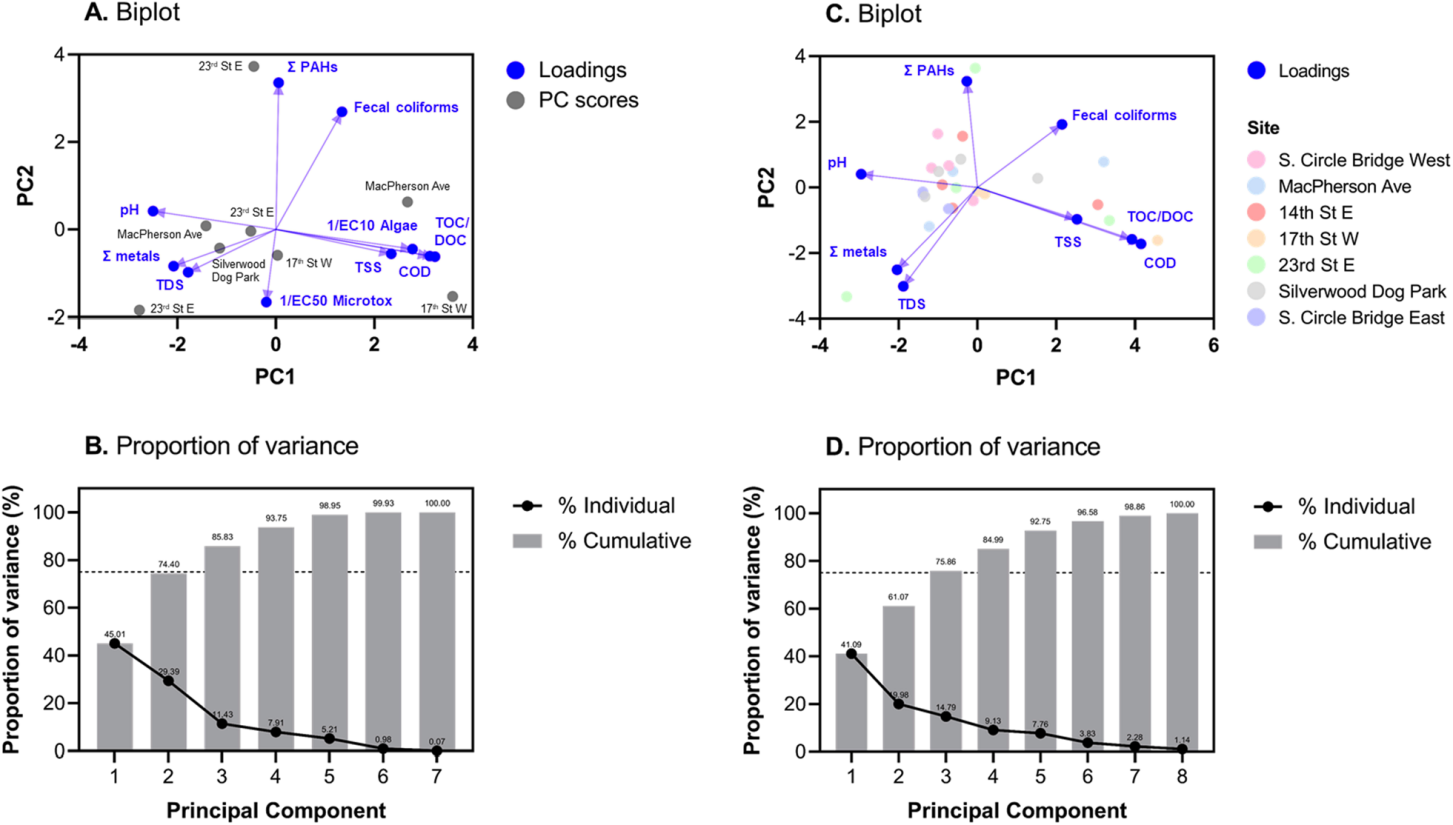
Principal component analysis (PCA) biplots and proportions of variance for two scenarios including: **A** and **B**: pH, TDS, TOC/DOC, COD, fecal coliforms, TSS, sum of metals, sum of PAHs, 1/EC_10_ (algae), and 1/EC_50_ (Microtox) (Table 2; Additional file 1: Tables S5, S7, and S8); and **C** and **D**: pH, TDS, TOC/DOC, COD, fecal coliforms, TSS, and sum of metals (Additional file 1: Tables S5, S7, and S8). The dotted lines in **B** and **D** indicate the typical cut-off of 75% for total variance used in PCA

Results of the PCA for study sampling sites and dates including physicochemical parameters pH, TDS, TOC/DOC, COD, fecal coliforms, TSS, sum of metals, and sum of PAHs are presented in Fig. 4C and D. The first two principal components (PCs) accounted for 61.1% which was below the 75% typical recommended minimum (Fig. 4B); however, this was deemed adequate for the current discussion given the PCA with toxicity data included only PC1 and PC2 (74.4% of variance) (Fig. 4B) which will be discussed in “Principal component analysis including toxicity data” section. The PC scores for the seven individual sampling locations and dates indicated no correlations between sites as scores were scattered throughout the PC1 and PC2 range (Fig. 4C). This is consistent with discussion within “pH, total dissolved solids (TDS), and electrical conductivity (EC)”, “Dissolved organic carbon (DOC), chemical oxygen demand (COD), and total suspended solids (TSS)” and “Coliform analysis” sections and for the individual physicochemical parameter univariate analysis which showed high variabilities between both individual sites and dates of sampling events. Of more interest are the biplot loadings (Fig. 4C) which indicate physicochemical parameters associated with PC1 and PC2. The TOC/DOC, COD, TSS, and fecal coliforms were positively associated with PC1, while pH, sum of metals, sum of PAHs (marginally negative), and TDS were negatively associated with PC1. For PC2, the pH, sum of PAHs, and fecal coliforms exhibit positive loadings with the remaining parameters having negative correlations.

The grouping of TOC/DOC, COD, and TSS would be expected based on discussion presented in “Dissolved organic carbon (DOC), chemical oxygen demand (COD), and total suspended solids (TSS)” section. In addition, fecal coliforms have been previously shown to be correlated with suspended solids as they can be bond to these solids in the environment [45]. The grouping of pH, sum of metals, and TDS would also be expected as lower pH leads to increased metal solubilities. For example, Johansson et al. [40] showed metal concentrations increased 2–5 times with a decrease in pH from 7.0 to 6.2–6.5 in river water. In addition, higher TDS results in higher conductivity and lower pH as shown previously (Islam et al. 2017). The only anomalous result are the PAHs which would also be expected to be associated with the TOC/DOC, COD, and TSS grouping as PAHs are typically bond to. For example, PAHs have been shown to be sorbed to natural sediments and other organic fractions previously [79]. However, this may be an artifact of the sample processing procedure for PAHs in which the filtration using a 0.45 µm filter may lead to the inaccurate reporting of total PAH concentrations. Future work will include consideration of the PAHs associated with particulates for an improved understanding of their loadings. However, the consideration of filtered PAHs is currently of interest for the toxicity assessment presented in the following section as the passing 0.45 µm PAHs would be the ‘freely dissolved’ portion which is expected to be readily available to organisms in the environment.

### Toxicity assessment

#### Algae bioassay

All samples were filtered (see “Methods” section) prior to testing, therefore all particle-bound contaminants were removed prior to testing. The 72-h growth rate of *R. subcapitata* satisfied the test validity requirements of the EPA method. Of the 16 samples tested for algal growth inhibition, 5 produced a toxic response (Table 2). Most samples did not inhibit algal growth by more than 20% at full concentration and all concentrations in the diluted series stimulated similar growth to negative controls. Most toxic samples were from the June 12 event, with one each from the July 25 and August 22 events. All samples originated from different outfalls; however, samples from the 17th St. W. outfall exhibited an IC_50_ below 33% concentration on both June 12 and August 22. Interestingly, the August 22 Silverwood Dog Park sample did not induce a toxic response, though it contained nearly twice the concentration of zinc from the outfall’s response-inducing July 25 sample. Pearson’s correlations between the IC_10_ and DOC, chloride, and individual metals and PAHs, respectively, only found correlations for cadmium (*r* = 0.693).

The acute toxicity of PAHs to green algae and daphnids has been previously correlated to the amount of LMW, hydrophilic PAHs in an aqueous mixture [14]; however, no correlation was observed in either species in this study. Similarly for metals, despite copper and zinc are primary causes of toxicity in aquatic organisms [5, 34, 52], no correlation was observed in this study (*r* = − 0.190 and *r* = 0.369, respectively). Previously reported IC_50_ and IC_10_ concentrations for *R. subcapitata* used SW with various contaminant concentrations one to two orders of magnitude larger than in this study [14]. Despite these elevated concentrations, Bragin et al. [14] also, however, noted the effects of PAHs on algae growth were limited. In this study, correlations between algae EC_10_ and contaminant concentration was only observed for cadmium, though concentrations of copper and zinc regularly exceeded CCME guidelines. As *R. subcapitata* can acclimate to aquatic concentrations between 0.5 and 100 µg Cu/L [13], local algae species may accommodate higher geogenic background levels. Brix et al. [15] identified relatively low zinc toxicity risk for brief, one-hour events and relatively significant toxicity for chronic exposures, which could explain the lack of toxicity despite a seasonal average of 237.5 µg/L. Another explanation for lack of toxicity may be due to the high TDS (and DOC) content, which will reduce metals bioavailability through competition and ligand formation. The antagonistic effects of multiple metals have been observed in this species: with respect to the presence of cadmium, significant cell density decrease has been observed in *R. subcapitata* [63]; however, when exposed to a mixture of copper, cadmium, nickel, and zinc, significant antagonistic interactions were observed in the organism [58]. This is the most likely explanation for the general lack of toxicity observed in the dissolved SW fraction.

#### Microtox bioassay

Microtox results did not indicate significant toxicity trends in *V. fischeri* exposed to filtered SW samples. Generally, samples diluted 50% or greater were not significantly inhibited relative to the negative control. All samples (except the MacPherson Ave. July 25 sample) showed some luminescence inhibition relative to the negative control after 15 min. As expected for SW, toxicity characteristics were highly variable within samples. As a defined toxicity trend was not observed in pre-filtered SW samples, it is likely the bulk of contaminants were particle-bound and remained in sample sediment. Though potential correlations between IC_10_ and DOC, chloride, and individual metals and PAHs were examined, Pearson’s correlations were only observed between the IC_10_ and aluminum (*r* = 0.514) or DOC (*r* = 0.524) concentrations.

In testing binary mixtures of metals Fulladosa et al. [24, 25], found copper–zinc mixtures to be additively toxic while Tsiridis et al. [74] observed a synergistic effect. Interestingly, the latter comments that all copper–zinc IC_50_ results differed significantly from theoretical predictions; when combined with mixtures of humic acids, the toxicity of the solution decreased relative to the metals-only mixture. As observed in the response of *R. subcapitata* to SW, the presence of TDS and DOC may have reduced the bioavailability of contaminants. Based on the literature and lack of a defined toxic response, it is inferred the overall mixture of metals and organic contaminants in tested SW samples produces an overall antagonistic effect relative to any single metal or PAH species: while copper and zinc concentrations in this study exceed the single-species IC_50_ concentrations for *V. fischeri* reported by Hsieh et al. [35], other studies observed 15-min IC_50_ values at greater concentrations of varying orders of magnitude, even in the additive or synergistic mixtures [24, 25, 28, 66, 74, 75].

#### Principal component analysis including toxicity data

Results of the PCA for study sampling sites and dates including physicochemical parameters pH, TDS, TOC/DOC, COD, fecal coliforms, TSS, sum of metals, sum of PAHs, 1/EC_10_ (algal toxicity), and 1/EC_50_ (Microtox toxicity) are presented in Fig. 4A and B. The first two PCs accounted for 74.4% of the variance which was considered to be reasonable for the current discussion following results without toxicity presented in “Principal component analysis excluding toxicity data” section. Overall, the results for the other physicochemical parameters showed similar loadings both with and without consideration of the toxicity assays which is expected considering the toxicity is a result of these other physicochemical parameters, thus, should not impact their distributions and behaviors in the environment. Interestingly, the algal and Microtox toxicity were positively and negatively associated with PC1, respectively. In contrast, both toxicities were negatively associated with PC2. The positive correlation (i.e., reduction in toxicity) of algal toxicity with solids would be expected based on the previous discussion as less metals and PAHs would be bioavailable, thus toxic to algal, in the presence of elevate organics for sorption. However, Microtox results show limited correlations with other physicochemical parameters as compared with the algal toxicity results. Although results for toxicity are of interest currently, the limited dataset may be the issue with the lack of Microtox correlations with other parameters.

### Land use management

Land use classification data based on Additional file 1: Figure S2 are presented in Table 3. The current study catchments comprise 33.5% of the CoS area with a total of 76.3 km^2^. The two most dominant land uses in the CoS are single-family residential (SR) at 29.9% and green (GR) at 26.9% (Table 3). Industrial (IN) is the next largest usage at 14.1%, while the remaining uses all fall below 10% including multi-family residential (MR; 5.42%), roads (R; 5.38%), highways (HW; 4.17%), commercial (CM; 7.62%), and agricultural (AG; 3.74%). However, the individual distribution of land uses for each catchment differed markedly making each unique in their potential contribution to the loading of contaminants into the SSR These individual percentages were used, along with data from Additional file 1: Tables S2 and S3, and the loading formula provided in the Methods, to determine summer 2019 seasonal contaminant loadings for TSS, COD, copper, nickel, lead, zinc, and PAHs as shown in Additional file 1: Table S9. These theoretical values were compared on a catchment scale to measured loading estimates using the average seasonal concentration of analyzed contaminants. The results are presented in Table 4. Compared to theoretical estimates, the loading of TSS and COD were higher and dissolved metals were lower in this study (PAHs were comparable with a few elevated pulses observed relative to the theoretical; see Table 4). The large discrepancy in lead concentration is likely due to some theoretical SMC sources predating the phase-out of leaded gasoline. Continuous flow (and the prevention of tunnel particulate accumulation) likely explains the TSS estimate for the SCB E outfall, which was threefold lower than theoretical as opposed to slightly higher. Catchment loading analysis below is based on the theoretical estimates, as these are established EMC values as opposed to the average of a small set of grab-sampling points.

**Table 3:** Land use classifications of City of Saskatoon (CoS) catchments included in this study

Catchment name	Area (km^2^)	CoS (%)	SR (%)	MR (%)	R (%)	HW (%)	CM (%)	IN (%)	GR (%)	AG (%)
Silverwood Dog Park	25.5	11.2	0	0	4	4	0	37	55	0
Circle Dr S Bridge W	24.6	10.8	35	10	9	7	7	15	17	0
Circle Dr S Bridge E	9.58	4.20	25	5	8	8	9	25	20	0
17th St. W.	9.27	4.06	39	16	8	5	14	5	5	0
14th St. E.	3.18	1.39	54	12	9	4	10	0	8	3
23rd St. W.	2.73	1.20	20	6	10	6	29	25	4	0
MacPherson Ave	1.47	0.64	54	12	9	4	10	0	8	3
Total	76.3	33.5	29.9	5.42	5.38	4.17	7.62	14.1	26.9	3.74

Catchment area data were provided by the CoS

*SR* single-family residential; *MR* multi-family residential; *R* roads; *HW* highways; *CM* commercial; *IN* industrial; *GR* green space; *AG* agricultural use

Represented as percentage of land use over total area of the CoS, as shown by labeled catchments in Additional file 1: Figure S1

**Table 4:** Comparison of measured versus theoretical loadings per catchment for the sampled 2019 ice-free SW season (June–August)

	Land use area (km^2^)	Seasonal SW volume (10^3^ m^3^)	Measured seasonal loading (kg)							Theoretical seasonal loading (kg)						
			TSS	COD	Cu	Ni	Pb	Zn	PAHs	TSS	COD	Cu	Ni	Pb	Zn	PAHs
Wanuskewin Rd	25.5	1216	264,330 ± 211,021	565,293 ± 450,796	48.9 ± 50.5	8.15 ± 14.1	1.55 ± 1.61	546 ± 120	0.77 ± 0.52	345,189 ± 250,796	156,735 ± 67,511	121 ± 81.6	39.2 ± 12.4	224 ± 204	428 ± 294	1.09 ± 0.31
SCB E	24.6	1567	350,679 ± 197,142	443,541 ± 498,276	68.5 ± 76.1	13.6 ± 23.5	2.66 ± 1.76	375 ± 200	3.10 ± 3.73	362,769 ± 258,577	167,340 ± 71,554	109 ± 68.0	41.3 ± 14.9	214 ± 193	453 ± 313	1.33 ± 0.34
SCB W	9.58	757	38,158 ± 22,205	124,225 ± 97,227	54.0 ± 55.7	1.39 ± 1.43	3.55 ± 2.31	143 ± 80.6	0.59 ± 0.38	194,226 ± 138,684	89,451 ± 36,241	61.8 ± 35.6	21.3 ± 8.76	119 ± 106	239 ± 173	0.69 ± 0.04
17th St. W.	8.99	697	330,369 ± 286,392	655,420 ± 611,534	26.7 ± 17.1	5.13 ± 5.57	1.49 ± 0.73	118 ± 25.3	0.43 ± 0.46	125,815 ± 89,836	60,563 ± 24,537	38.5 ± 22.2	15.3 ± 6.3	74.5 ± 66.2	165 ± 119	0.47 ± 0.03
14th St. E.	3.18	223	53,097 ± 43,049	109,903 ± 121,122	7.58 ± 7.84	1.05 ± 1.24	0.42 ± 0.16	42.4 ± 12.1	0.17 ± 0.15	45,586 ± 32,256	22,174 ± 9,280	13.0 ± 7.84	5.64 ± 2.08	25.6 ± 23.1	58 ± 40.2	0.17 ± 0.02
MacPherson Ave	1.47	103	23,642 ± 22,043	60,653 ± 69,697	4.25 ± 4.84	0.52 ± 0.72	0.14 ± 0.05	22.0 ± 9.29	0.05 ± 0.7	21,073 ± 15,614	10,250 ± 4,724	6.0 ± 4.24	2.61 ± 0.75	11.9 ± 11.0	27 ± 17.7	0.08 ± 0.00
23rd St. W.	2.73	259	135,986 ± 110,662	160,338 ± 204,452	13.9 ± 15.4	3.54 ± 6.13	0.53 ± 0.24	54.9 ± 38.6	0.47 ± 0.17	63,584 ± 47,564	29,467 ± 11,253	21.7 ± 16.1	7.26 ± 1.23	40.5 ± 37.3	79 ± 56.9	0.22 ± 0.14

Measured seasonal loading was calculated by averaging measured concentrations of the indicated parameters to obtain a seasonal mean concentration per catchment. Seasonal SW volumes were calculated from local rain gauge data (see “Methods” and “Land use management” sections). Mass loading values were obtained using estimated seasonal runoff and compared to the sums of theoretical catchment loads. Parameters were selected for direct comparison with theoretical values

Based on the average of measured concentrations over sampled events, SCB W catchment produced the greatest seasonal loading for all parameters at approximately 351,000 kg, 444,000 kg, 68.5 kg, 13.6 kg, 2.66 kg, 375 kg, and 3.10 kg, respectively. These values represent 21–56% of the total study loadings of 1,200,000 kg, 2,100,000 kg, 224 kg, 33.3 kg, 10.3 kg, 1301 kg and 5.58 kg, respectively. As the SCB W catchment comprises 32% of the study area, these loadings are roughly proportional to the land use area but vary greatly; the trend is similar for the Wanuskewin Rd. catchment, at 14–42% of total study catchment loading and 34% of total study area. The slightly smaller proportion of loading estimated from the Silverwood catchment is due to lesser depth of precipitation observed at local rain gauges relative to the SCB W catchment. Though the two largest catchments, Wanuskewin Rd. and SCB W, differ greatly in land use (Table 2), they are similar in area; other study catchments contribute loading generally proportional to their surface area as a percent of the CoS. This agrees with previous observations that catchment area is the dominant driver of contaminant loading [3].

Inputs from roads, highways, and commercial or industrial land uses contribute significantly to high loadings. Industrial land use was most strongly correlated with TDS, TSS, COD, and Ni loading while multi-residential land use was most strongly correlated with Cu, Pb, Zn, and PAH loadings (Additional file 1: Table S10). Incidentally, single-residential land use was almost as strongly correlated with TDS as industrial land use. Residential land use loadings may be attributed to the density of driveways and parked vehicles in these land uses. The industrial land use in the Wanuskewin Rd. catchment is estimated to contribute 8.6–26% of loading from all 7 study catchments despite comprising 12% of the area. The SCB E catchment, with the second-greatest industrial land use, contributes 3.2–34% of total study catchment loading from 12% of the area, with proportionally low TSS and high lead discharges. In the 23rd St. W. catchment, located in the city center, commercial and industrial land use also dominate pollutant loadings, though road loading of COD and nickel were comparable to loading from industrial land use. Conversely, in residential-dominant areas, roads tend to comprise the largest input of COD, TSS, nickel, and lead. For example, roads and highways in the Light & Power catchment are each one-third the surface area of single-residential areas, yet each contribute almost 300% more COD and TSS loading (industrial land use generates twice as much per km^2^). Al Masum et al. [3] previously observed the impact of land use on CoS contaminant loading in 2017, also noting that industrial areas had the highest relative loadings.

The first flush has been correlated with significantly increased TSS concentration [8] and 25–95% contaminant removal in the first 50% of runoff volume [42]. The tracing of pollutant loading across three storm events by Zhang et al. [82] indicate a high first flush effect across COD, TN, TP, TSS, and dissolved copper and zinc. The study clearly delineated both rainfall and contaminant peaks, accomplished using intensive sampling at 5-min intervals for the first 60 min of the storm. Deletic [20] previously remarked the relatively low sampling resolutions would limit insight into the first flush behavior of brief, intense, 30-min storms, which are common on the Canadian Prairies. Grab sampling indeed limited the ability of this study to interpret SW data with respect to an event volume or contamination peak. Passive sampling strategies could be used to improve the quality of the data, allowing datasets comparable to Zhang et al. [82] to be created. The use of passive sampling equipment would allow multiple samples at multiple outfalls to be simultaneously collected close to the onset of runoff. The addition of flow rate measurement at the time of each sample collection would allow event-specific contaminant and runoff curves to be extrapolated and minimize uncertainty associated with the current grab sampling method. It is nonetheless obvious that the July 25 event, sampled hours after the storm as opposed to its onset, contains significantly less COD and TSS relative to other events.

While much of the TSS in these samples can be attributed to the tarring, the antecedent dry period likely also plays a role. Dry weather was observed from the July 25 event until the August 22 event, contributing to elevated pollutant loading for all catchment areas. Though the June 20 storm was the first major (> 10 mm rainfall) event of the season, its TSS loading is comparable to the July 10/18 (19 mm depth reported by the CoS) and August 22 events; hourly rainfall gauge data indicates a first flush may have been missed 4 h prior to sampling. First flush is not a universally observed phenomenon and is influenced by land use and local topography: assuming a majority of the pollutant mass is transported in the first volume of SW (specific author definitions differ), first flushes are often not found distinctive across TSS, EC, pH, and temperature [9, 20, 70]. Many studies note nonetheless that land use and storm characteristics induce a first flush effect for some contaminants such as dissolved metals [70]. Due to these small-scale influences within the catchment, modeling street-level runoff and sewer flow behavior may be the best method for estimating the catchment-wide frequency, magnitude, and spatiotemporal distribution of the first flush.

## Conclusions

Stormwater (SW) samples were obtained across four 2019 summer storm events from seven major catchments within the City of Saskatoon (CoS). Initial SW quality data were obtained for runoff outlets discharging from 33% of CoS urban surface area, including industrial and downtown commercial districts. Though sampling data are limited to single data points per event, this study provides preliminary identification of the nature of contamination present in each catchment and establishes a point of reference from which data quality may be improved. Seasonal trends could not be concluded from this study as scarce storm events over the sampling season severely limited sampling opportunity. Catchment-related activity was observed to drive variation between event-related datasets.

In general, stormwater characteristics were similar to those of previous studies, with a bulk of contamination carried by the first volume of runoff, influenced by a combination of rainfall depth, antecedent dry period, land use, and activity within the catchment. The most significant SW quality variation between events was due to road tarring activity occurring within the city prior to the August 22 event. Seasonal trends were not observed over the summer; climate conditions over the course of the study included extended dry periods and few summer storms. A lack of distinctive toxicity in filtered samples, along with comparable dissolved metals and PAH values across all events, indicates much of the contamination is particle-bound, furthermore, the overall mixture of contaminants likely antagonizes the toxic mechanism of any given contaminant species. The catchment area was observed to be a dominant driver of loading; however, grab sampling strategies prohibited assessing the influence of rainfall depth or intensity on contaminant loading. More intensive sampling strategies are necessary to contextualize stormwater data in the context of contaminant and runoff volume peaks; furthermore, intensive sampling data will create more robust flow and transport models. However, this research nonetheless adds to the existing literature on cold-climate, semi-arid stormwater, contributes to local environmental quality data, and provides a preliminary snapshot into local stormwater characteristics.

## Supplementary Information

**Additional file 1: Table S1.** Limits of detection (LOD) and quantification (LOQ) for polyaromatic hydrocarbons. **Table S2.** Land use classifications used to calculate runoff volumes in this study based on Järveläinen et al. [39]. The runoff coefficients (CR) considered in the current study follow City of Saskatoon (CoS) stormwater management guidelines. **Table S3. **Average flow-weighted site mean concentration (SMC) values for different land use classes, from Melanen (1981), Mitchell (2005), Nordeidet et al. (2004) and Järveläinen et al. [39] as adapted by Al Masum et al. [3]. The SMC is the geometric mean of the event mean concentration for each storm event, which is the concentration of pollutants as a function of the runoff volume discharging in the river (flow-weighted). The value is used to estimate overall SW contaminant loading over a given urban area. Values in parentheses are standard deviations (SD). **Table S4**. Overview of analyzed stormwater quality parameters for the 2019 sampling season grouped by site. Catchments outlined in Additional file 1: Figure S1. Values are average (standard deviation, SD). Quality parameter abbreviations are as follows: total dissolved solids (TDS), electrical conductivity (EC), dissolved organic carbon (DOC), chemical oxygen demand (COD), and total suspended solids (TSS). **Table S5.** Overview of stormwater quality parameters for the 2019 sampling season for each individual event and outfall. Catchments outlined in Additional file 1: Figure S1. **Table S6.** Chloride and sulphate analysis for select 2019 stormwater samples. **Table S7. **Metals (µg/L) detected in 2019 stormwater samples. **Table S8. **PAHs (ng/L) detected in 2019 stormwater samples. *NM* not measured. **Table S9. **Theoretical seasonal loading estimates for various physicochemical parameters of interest. Estimates in this table are based on theoretical SMC values given in Additional file 1: Table S3. Rainfall depths used to estimate seasonal catchment runoff volumes are included in Additional file 1: Table S4. Estimates using measured seasonal mean concentrations are included in Additional file 1: Table S10. **Table S10.** Correlations between runoff volume from land use area across each catchment (calculated from surface area; see “Methods” section) and contaminant loading. Loading is based upon averages of measured 2019 SW concentrations; see Additional file 1: Table S5. **Figure S1.** Stormwater catchments delineated within the CoS following Al Masum et al. [3]. Catchments included in this study are Light & Power (SCB E), Circle Drive S Bridge (SCB W), 14th St. E. (including both the MacPherson Ave. and 14th St. E. catchments), 17th St. W., 23rd St. W., and Wanuskewin Road (Silverwood Dog Park). Stormwater sampling occurred over summer 2019; the data are intended to complement a parallel 2018 study examining summer SW quality in the Taylor Street, Avenue B S, Preston Crossing, Dog Park (Preston), Spadina/Sturgeon, and Whiteswan/WWTP catchments.** Figure S2.** Land use breakdown of study catchments. The 14th St. E. catchment above drains to both the MacPherson Ave. and 14th St. E. outfalls (though not delineated on the land-use map, areas for each subcatchment are provided by the CoS). Adapted from Al Masum et al. [3]. Refer to Additional file 1: Table S1 for land use classification acronyms. Refer to Additional file 1: Table S2 for total and land-use surface area in km^2^.

## Data Availability

Not applicable.
