# Supplementary material for: Assessment of stormwater discharge contamination and toxicity for a cold-climate urban landscape
Source: Environ Sci Eur. 2022 May 13;34(1):43. doi: 10.1186/s12302-022-00619-x (PMC9106602; doi:10.1186/s12302-022-00619-x)

**Supporting Information**

**ASSESSMENT OF STORMWATER QUALITY DISCHARGING FROM A COLD-CLIMATE URBAN LANDSCAPE DURING THE ICE-FREE SEASON**

Popick, H. ^1^, Brinkmann, M.^2,3,4,5^, McPhedran, K. ^1,2,*^

^1^ Department of Civil, Geological, and Environmental Engineering, College of Engineering, University of Saskatchewan, Saskatoon, SK, Canada

^2^ Global Institute for Water Security, University of Saskatchewan, Saskatoon, SK, Canada

^3^ Toxicology Centre, University of Saskatchewan, Saskatoon, SK, Canada

^4^ School of Environment and Sustainability, University of Saskatchewan, Saskatoon, SK, Canada

^5^ Centre for Hydrology, University of Saskatchewan, Saskatoon, SK, Canada

**Pages: 12**

**Tables: 9**

**Figures: 2**

**Table S1.** Limits of detection (LOD) and quantification (LOQ) for polyaromatic hydrocarbons.

| **Sample** | **Acenaphthylene** | **Fluorene** | **BbF** | **BkF** | **BaP** | **Benzo[a]**  **anthracene** | **Chrysene** | **Dibenzo[a,h]**  **anthracene** | **Pyrene** | **Phenanthrene** | **Antrhacene** | **Indeno[123]cd-pyrene** | **Benzo[ghi]**  **perylene** |
| --- | --- | --- | --- | --- | --- | --- | --- | --- | --- | --- | --- | --- | --- |
| LOD(ppb) | 0.004 | 0.002 | **0.004** | **0.006** | **0.006** | **0.002** | **0.004** | **0.012** | **0.004** | **0.004** | **0.004** | **0.004** | **0.006** |
| LOQ(ppb) | 0.02 | 0.02 | **0.026** | **0.034** | **0.032** | **0.014** | **0.022** | **0.074** | **0.022** | **0.02** | **0.022** | **0.03** | **0.042** |

####

#### Table S2. Land use classifications used to calculate runoff volumes in this study based on Järveläinen et al. (2017). The runoff coefficients (CR) considered in the current study follow City of Saskatoon (CoS) stormwater management guidelines.

| **Land use class** |  | **Acronym** | **CR** | **Description** |
| --- | --- | --- | --- | --- |
| Single-family Residential |  | SR | 0.3 | Single dwelling house |
| Multi-family Residential |  | MR | 0.6 | Multiple separate housing units within a building |
| Roads |  | R | 0.95 | With average traffic of fewer than 15,000 vehicles/day |
| Highways |  | HW | 0.95 | With average traffic of more than 15,000 vehicles/day |
| Commercial |  | CM | 0.6 | Downtown, central business district, shopping centre, university, hospital, etc. |
| Industrial |  | IN | 0.6 | Industrial area |
| Green |  | GR | 0.1 | Parks, forests, meadows, and undeveloped area |
| Agricultural |  | AG | 0.05 | Cultivated area |

**Table S3.** Average flow-weighted site mean concentration (SMC) values for different land use classes, from Melanen (1981), Mitchell (2005), Nordeidet et al. (2004) and Jarvelainen et al. (2017) as adapted by Al Masum et al. (2021). The SMC is the geometric mean of the event mean concentration for each storm event, which is the concentration of pollutants as a function of the runoff volume discharging in the river (flow-weighted). The value is used to estimate overall SW contaminant loading over a given urban area. Values in parentheses are standard deviations (SD).

| **Land use class** | **TSS (mg/L)** | **COD (mg/L)** | **Pb (μg/L)** | **Zn (μg/L)** | **Cu (μg/L)** | **Cr (μg/L)** | **Ni (μg/L)** | **PAHs (μg/L)** |
| --- | --- | --- | --- | --- | --- | --- | --- | --- |
| Single-family Residential (SR) | 100 (81) | 57 (36) | 60 (50) | 144 (109) | 32 (17.5) | 4.9 (3.5) | 16.5 (19) | 0.4 (0) |
| Multi-family Residential (MR) | 118 (47) | 68 (45) | 73 (71) | 252 (87) | 39.5 (8) | 13 (8) | 21 (13) | 0.6 (0) |
| Roads (R) | 271 (175) | 120 (62) | 114 (117) | 237 (42) | 55 (16) | 20 (17) | 29 (2) | 0.8 (0) |
| Highways (HW) | 288 (148) | 117 (70) | 166 (139) | 327 (173) | 64 (31) | 10 (4) | 15 (21) | 1.4 (0) |
| Commercial (CM) | 194 (160) | 91 (61) | 145 (140) | 260 (136) | 84 (71) | 14 (9) | 24 (8) | 0.6 (0) |
| Industrial (IN) | 194 (160) | 91 (61) | 145 (140) | 260 (136) | 84 (71) | 14 (9) | 24 (8) | 0.6 (0) |
| Green (GR) | 84 (61) | 38 (2.8) | 35 (30) | 116 (110) | 19 (12.5) | 7 (0) | 15 (0) | 0 (0) |
| Agricultural (AG) | 84 (61) | 38 (2.8) | 35 (30) | 116 (110) | 19 (12.5) | 7 (0) | 15 (0) | 0 (0) |

**Table S4**. Overview of analyzed stormwater quality parameters for the 2019 sampling season grouped by site. Catchments outlined in Figure S1. Values are average (standard deviation, SD). Quality parameter abbreviations are as follows: total dissolved solids (TDS), electrical conductivity (EC), dissolved organic carbon (DOC), chemical oxygen demand (COD), and total suspended solids (TSS).

| **Sampling Site** | **pH** | **TDS (mg/L)** | **EC (μS/cm)** | **DOC (mg/L)** | **COD (mg/L)** | **TSS (mg/L)** | **Coliform  (*E. coli*/100mL)** |
| --- | --- | --- | --- | --- | --- | --- | --- |
| **SCB E** | 7.98 (0.30) | 738 (205) | 1476 (392) | 14.7 (2.8) | 164 (151) | 50.4 (46.4) | 158 (117) |
| **SCB W** | 8.44 (0.69) | 363 (108) | 745 (217) | 14.6 (9.9) | 283 (367) | 224 (145) | 2283 (2410) |
| **MacPherson Ave.** | 8.10 (1.54) | 349 (243) | 711 (485) | 69.5 (81.1) | 588 (532) | 229 (154) | 5963 (4919) |
| **14^th^ St. E** | 7.75 (1.55) | 389 (123) | 793 (243) | 96.1 (136) | 493 (531) | 238 (238) | 4070 (3719) |
| **17^th^ St. W** | 7.39 (1.76) | 283 (66.7) | 583 (137) | 116 (175) | 940 (1,196) | 474 (444) | 284 (490) |
| **23^rd^ St. E** | 8.30 (1.39) | 560 (499) | 1122 (969) | 55.7 (68.6) | 620 (822) | 526 (566) | 5000 (5772) |
| **Silverwood Dog Park** | 7.99 (1.26) | 285 (143) | 586 (290) | 10.3 (2.6) | 465 (684) | 217 (204) | 400 (464) |
| **AVERAGE (SD)** | **7.99 (0.35)** | **424(167)** | **859(327)** | **53.9 (42.7)** | **507 (250)** | **280 (164)** | **2594 (2432)** |

Table S5. Overview of stormwater quality parameters for the 2019 sampling season for each individual event and outfall. Catchments outlined in Figure S1.

| **Sampling Site Name And Date** | **pH** | **TDS (mg/L)** | **EC (μS/cm)** | **DOC (mg/L)** | **COD (mg/L)** | **TSS (mg/L)** | **Coliform (*E. coli*/100mL)** |
| --- | --- | --- | --- | --- | --- | --- | --- |
| **June 12, 2019** | | | | | | | |
| SCB E | 8.11 | 974 | 1914 | 17.2 | 117 | 13.5 | <1 |
| SCB W | 8.10 | 446 | 911 | 11.6 | 134 | 300.5 | <1 |
| MacPherson Ave. | 9.09 | 713 | 1438 | 60.1 | 748 | 171 | 3850 |
| 14^th^ St. E | 7.50 | 551 | 1110 | 60.3 | 362 | 24.5 | 1700 |
| 17^th^ St. W | 7.02 | 337 | 694 | 54.8 | 334 | 201.5 | <1 |
| 23^rd^ St. E | 9.52 | 1295 | 2550 | 37.8 | 242 | 138 | <1 |
| Silverwood Dog Park | 7.48 | 222 | 457 | 11.5 | 87.5 | 56 | <1 |
| **AVERAGE (SD)** | **8.12 (0.90)** | **648 (378)** | **1296 (733)** | **36 (23)** | **289 (229)** | **129 (105)** | **794(1,489)** |
| **June 20, 2019** | | | | | | | |
| SCB E | 8.13 | 570 | 1156 | 14.1 | 88 | 83.5 | 250 |
| SCB W | 9.46 | 445 | 909 | 8.3 | 116 | 183.5 | 400 |
| MacPherson Ave. | 9.26 | 222 | 459 | 16.4 | 217.5 | 376 | <1 |
| 14^th^ St. E | 9.56 | 417 | 853 | 12.4 | 243.5 | 526.5 | 100 |
| 17^th^ St. W | 9.14 | 301 | 620 | 12.5 | 590.5 | 1045 | 850 |
| 23^rd^ St. E | 9.02 | 359 | 736 | 19.1 | 292.5 | 583.5 | <1 |
| Silverwood Dog Park | 9.69 | 313 | 645 | 6.5 | 176 | 360 | <1 |
| **AVERAGE (SD)** | **9.18 (0.52)** | **375 (114)** | **768 (228)** | **12.8 (4.4)** | **246 (168)** | **451 (315)** | **272(313)** |
| **July 25, 2019** | | | | | | | |
| SCB E | 8.14 | 562 | 1135 | 10.9 | 62.5 | 7.5 | 245 |
| SCB W | 8.27 | 344 | 707 | 9.1 | 50.5 | 39.5 | 4420 |
| MacPherson Ave. | 8.14 | 226 | 467 | 14.5 | 118 | 39.5 | <9999 |
| 14^th^ St. E | 8.11 | 304 | 623 | 14.6 | 93.5 | 61.5 | 7295 |
| 17^th^ St. W | 8.29 | 186 | 384 | 20.3 | 124 | 55 | <1 |
| 23^rd^ St. E | 8.29 | 188 | 390 | 8.8 | 97.5 | 77 | <9999 |
| Silverwood Dog Park | 8.06 | 470 | 960 | 11.2 | 106 | 28.5 | 855 |
| **AVERAGE (SD)** | **8.19 (0.10)** | **326 (145)** | **667 (290)** | **13 (4)** | **93 (27)** | **44 (23)** | **4,688(4,467)** |
| **August 22, 2019** | | | | | | | |
| SCB E | 7.52 | 845 | 1700 | 16.4 | 388 | 97 | 135 |
| SCB W | 7.94 | 218 | 452 | 29.2 | 831 | 371.5 | 4310 |
| MacPherson Ave. | 5.92 | 233 | 481 | 187 | 1269 | 330.5 | <9999 |
| 14^th^ St. E | 5.82 | 283 | 584 | 297 | 1271 | 339.5 | 7,185 |
| 17^th^ St. W | 5.09 | 309 | 635 | 378 | 2711 | 593.5 | <1 |
| 23^rd^ St. E | 6.35 | 396 | 813 | 157 | 1847 | 1304 | <9999 |
| Silverwood Dog Park | 6.73 | 136 | 282 | 12.1 | 1490 | 425 | 745 |
| **AVERAGE (SD)** | **6.48 (1.00)** | **346 (235)** | **707 (468)** | **154 (145)** | **1401 (743)** | **494 (386)** | **4,625(4,491)** |

**Table S6.** Chloride and sulphate analysis for select 2019 stormwater samples.

| **Date** | **Sampling Site** | **Chloride (mg/L)** | **Sulphate (mg/L)** |
| --- | --- | --- | --- |
| June 12/19 | SCB E | 140 | 700 |
| June 12/19 | MacPherson Ave. | 170 | 23 |
| June 12/19 | 23^rd^ St. W | 94 | 130 |
| July 25/19 | SCB E | 82 | 370 |
| August 22/19 | SCB E | 220 | 310 |

|  | **Sample ID** | **Al** | **As** | **B** | **Ba** | **Br** | **Cd** | **Co** | **Cu** | **Fe** | **Mn** | **Mo** | **Ni** | **Pb** | **Sb** | **Sn** | **Sr** | **Th** | **Ti** | **U** | **V** | **Zn** |
| --- | --- | --- | --- | --- | --- | --- | --- | --- | --- | --- | --- | --- | --- | --- | --- | --- | --- | --- | --- | --- | --- | --- |
| June 12/19 | SCB E | 143 | 6 | 149 | 36 | 226 | 0 | 0 | 196 | 108 | 86 | 7 | 0 | 13 | 0 | 9 | 782 | 0 | 0 | 18 | 0 | 223 |
|  | SCB W | 100 | 5 | 44 | 64 | 0 | 0 | 0 | 127 | 66 | 15 | 6 | 0 | 2 | 0 | 10 | 307 | 0 | 0 | 4 | 0 | 162 |
|  | MacPherson Ave. | 49 | 12 | 139 | 22 | 0 | 0 | 2 | 115 | 69 | 28 | 8 | 8 | 1 | 2 | 16 | 58 | 0 | 3 | 1 | 4 | 102 |
|  | 14th St. E | 38 | 60 | 127 | 40 | 182 | 0 | 2 | 67 | 92 | 260 | 5 | 7 | 1 | 4 | 4 | 414 | 0 | 0 | 14 | 3 | 184 |
|  | 17th St. W | 61 | 63 | 93 | 36 | 302 | 3 | 2 | 150 | 186 | 261 | 5 | 6 | 3 | 5 | 21 | 371 | 0 | 3 | 6 | 3 | 200 |
|  | 23rd St. E | 43 | 420 | 117 | 35 | 183 | 1 | 1 | 114 | 125 | 27 | 108 | 0 | 3 | 12 | 13 | 231 | 0 | 0 | 4 | 3 | 125 |
|  | Silverwood Dog Park | 31 | 6 | 23 | 32 | 0 | 0 | 0 | 116 | 67 | 83 | 8 | 0 | 2 | 0 | 15 | 150 | 0 | 0 | 3 | 0 | 197 |
| June 20/19 | SCB E | 141 | 38 | 149 | 37 | 234 | 0 | 1 | 53 | 116 | 80 | 9 | 0 | 2 | 0 | 0 | 427 | 0 | 0 | 8 | 0 | 120 |
|  | SCB W | 126 | 8 | 20 | 15 | 0 | 16 | 0 | 11 | 178 | 36 | 6 | 0 | 0 | 0 | 0 | 55 | 0 | 4 | 1 | 2 | 112 |
|  | MacPherson Ave. | 115 | 4 | 41 | 12 | 0 | 0 | 0 | 14 | 151 | 32 | 4 | 0 | 1 | 0 | 0 | 29 | 0 | 4 | 1 | 2 | 127 |
|  | 14th St. E | 143 | 7 | 29 | 16 | 0 | 1 | 1 | 10 | 156 | 37 | 10 | 0 | 1 | 0 | 0 | 40 | 2 | 7 | 1 | 2 | 136 |
|  | 17th St. W | 84 | 4 | 30 | 21 | 355 | 0 | 1 | 18 | 114 | 40 | 5 | 7 | 1 | 0 | 0 | 106 | 0 | 3 | 1 | 2 | 180 |
|  | 23rd St. E | 88 | 15 | 55 | 23 | 342 | 1 | 1 | 8 | 119 | 60 | 4 | 5 | 1 | 0 | 0 | 161 | 0 | 3 | 1 | 2 | 140 |
|  | Silverwood Dog Park | 134 | 5 | 0 | 19 | 0 | 0 | 0 | 8 | 169 | 30 | 5 | 0 | 1 | 0 | 0 | 42 | 0 | 3 | 0 | 2 | 132 |
| July 25/19 | SCB E | 55 | 12 | 89 | 43 | 141 | 0 | 0 | 12 | 51 | 59 | 19 | 0 | 1 | 0 | 0 | 437 | 0 | 0 | 8 | 0 | 222 |
|  | SCB W | 44 | 13 | 47 | 28 | 0 | 0 | 0 | 11 | 63 | 22 | 34 | 0 | 1 | 0 | 0 | 218 | 0 | 0 | 5 | 0 | 238 |
|  | MacPherson Ave. | 69 | 4 | 58 | 23 | 0 | 0 | 0 | 14 | 64 | 73 | 7 | 0 | 1 | 0 | 0 | 139 | 0 | 0 | 3 | 0 | 408 |
|  | 14th St. E | 229 | 7 | 158 | 42 | 0 | 0 | 0 | 40 | 104 | 45 | 12 | 0 | 3 | 0 | 0 | 162 | 0 | 2 | 2 | 0 | 190 |
|  | 17th St. W | 125 | 5 | 57 | 24 | 0 | 0 | 0 | 26 | 106 | 56 | 7 | 0 | 2 | 0 | 0 | 131 | 0 | 2 | 3 | 0 | 156 |
|  | 23rd St. E | 83 | 26 | 73 | 27 | 0 | 0 | 0 | 14 | 128 | 42 | 58 | 0 | 2 | 0 | 0 | 119 | 0 | 0 | 2 | 0 | 119 |
|  | Silverwood Dog Park | 55 | 10 | 74 | 38 | 224 | 0 | 1 | 12 | 49 | 8 | 14 | 0 | 1 | 0 | 0 | 240 | 0 | 0 | 4 | 0 | 541 |
| August 22/19 | SCB E | 355 | 3 | 29 | 32 | 0 | 0 | 1 | 24 | 267 | 103 | 3 | 7 | 3 | 0 | 0 | 65 | 0 | 8 | 1 | 2 | 192 |
|  | SCB W | 244 | 5 | 334 | 83 | 3473 | 0 | 1 | 25 | 234 | 172 | 6 | 35 | 3 | 0 | 0 | 3463 | 3 | 8 | 14 | 0 | 446 |
|  | MacPherson Ave. | 248 | 3 | 154 | 34 | 169 | 0 | 1 | 23 | 353 | 165 | 3 | 12 | 3 | 0 | 0 | 183 | 0 | 11 | 1 | 2 | 218 |
|  | 14th St. E | 181 | 4 | 88 | 32 | 0 | 10 | 1 | 19 | 278 | 195 | 5 | 12 | 2 | 0 | 0 | 109 | 0 | 13 | 1 | 4 | 251 |
|  | 17th St. W | 161 | 3 | 140 | 32 | 0 | 1 | 2 | 21 | 403 | 292 | 4 | 41 | 2 | 0 | 0 | 77 | 0 | 8 | 1 | 3 | 314 |
|  | 23rd St. E | 290 | 3 | 69 | 37 | 0 | 1 | 1 | 18 | 480 | 201 | 4 | 25 | 3 | 0 | 0 | 82 | 0 | 10 | 1 | 2 | 291 |
|  | Silverwood Dog Park | 224 | 9 | 10 | 23 | 0 | 10 | 1 | 23 | 304 | 51 | 3 | 27 | 2 | 0 | 0 | 45 | 0 | 9 | 0 | 2 | 924 |
|  | CCME Guideline | 100 | 5 |  |  |  | 0.37 |  | 2 | 300 |  | 73 | 25 | 1 |  |  |  |  |  | 15 |  | 30 |

**Table S7.** Metals (µg/L) detected in 2019 stormwater samples.

**Table S8.** PAHs (ng/L) detected in 2019 stormwater samples. NM = not measured.

|  | **Sample Id** | **Acenaphthylene (ACY)** | **Fluorene (FL)** | **Benzo[b] fluoranthene (BbF)** | **Benzo[k] fluoranthene (BkF)** | **Benzo[a] pyrene (BaP)** | **Benz[a] anthracene (BaA)** | **Chrysene (CHR)** | **Dibenz[a,h] anthracene (DahA)** | **Pyrene (PYR)** | **Phenanthrene (PHE)** | **Anthracene (ANT)** | **Indeno[1,2,3] cd-pyrene (I123P)** | **Benzo[g,h,I] perylene (BghiP)** | **SUM** |
| --- | --- | --- | --- | --- | --- | --- | --- | --- | --- | --- | --- | --- | --- | --- | --- |
| June 12/19 | SCB E | NM | NM | NM | NM | NM | NM | NM | NM | NM | NM | NM | NM | NM | NM |
|  | SCB W | 0.000 | 0.000 | 0.000 | 0.000 | 0.949 | 0.265 | 0.269 | 0.198 | 0.934 | 1.386 | 1.399 | 0.293 | 0.388 | 6.079 |
|  | MacPherson Ave. | 0.066 | 0.000 | 0.113 | 0.088 | 0.135 | 0.097 | 0.112 | 0.056 | 0.046 | 0.000 | 0.000 | 0.095 | 0.082 | 0.890 |
|  | 14th St. E | 0.000 | 0.000 | 0.000 | 0.000 | 0.000 | 0.000 | 0.000 | 0.074 | 0.000 | 0.000 | 0.000 | 0.075 | 0.034 | 0.183 |
|  | 17th St. W | 0.056 | 0.034 | 0.000 | 0.000 | 0.000 | 0.027 | 0.011 | 0.015 | 0.034 | 0.029 | 0.025 | 0.022 | 0.012 | 0.264 |
|  | 23rd St. E | 0.000 | 0.000 | 0.000 | 0.000 | 0.000 | 0.020 | 0.024 | 0.017 | 0.000 | 0.000 | 0.000 | 0.025 | 0.012 | 0.099 |
|  | Silverwood Dog Park | 0.472 | 0.319 | 0.026 | 0.026 | 0.026 | 0.014 | 0.005 | 0.011 | 0.032 | 0.016 | 0.028 | 0.017 | 0.010 | 0.999 |
| June 20/19 | SCB E | 0.000 | 1.147 | 0.000 | 0.000 | 0.000 | 0.034 | 0.039 | 0.024 | 0.056 | 0.061 | 0.075 | 0.027 | 0.017 | 1.479 |
|  | SCB W | 0.523 | 0.000 | 0.051 | 0.051 | 0.069 | 0.051 | 0.033 | 0.044 | 0.051 | 0.032 | 0.027 | 0.041 | 0.030 | 1.002 |
|  | MacPherson Ave. | 0.000 | 0.000 | 0.000 | 0.000 | 0.000 | 0.020 | 0.024 | 0.023 | 0.039 | 0.035 | 0.047 | 0.018 | 0.027 | 0.233 |
|  | 14th St. E | 0.000 | 0.000 | 0.030 | 0.022 | 0.050 | 0.012 | 0.007 | 0.014 | 0.068 | 0.057 | 0.069 | 0.289 | 0.016 | 0.633 |
|  | 17th St. W | 0.000 | 0.000 | 0.000 | 0.000 | 0.050 | 0.018 | 0.015 | 0.000 | 0.031 | 0.028 | 0.017 | 0.023 | 0.011 | 0.192 |
|  | 23rd St. E | 0.000 | 0.227 | 0.000 | 0.000 | 0.041 | 0.012 | 0.009 | 0.000 | 0.038 | 0.023 | 0.035 | 0.026 | 0.022 | 0.433 |
|  | Silverwood Dog Park | 0.000 | 0.000 | 0.058 | 0.053 | 0.081 | 0.055 | 0.051 | 0.044 | 0.042 | 0.027 | 0.039 | 0.037 | 0.058 | 0.544 |
| July 25/19 | SCB E | 0.349 | 0.087 | 0.000 | 0.000 | 0.000 | 0.028 | 0.033 | 0.033 | 0.000 | 0.042 | 0.057 | 0.033 | 0.019 | 0.680 |
|  | SCB W | 0.102 | 0.102 | 0.000 | 0.000 | 0.000 | 0.000 | 0.000 | 0.000 | 0.030 | 0.023 | 0.033 | 0.021 | 0.013 | 0.325 |
|  | MacPherson Ave. | NM | NM | NM | NM | NM | NM | NM | NM | NM | NM | NM | NM | NM | NM |
|  | 14th St. E | 0.000 | 0.793 | 0.000 | 0.012 | 0.125 | 0.000 | 0.000 | 0.008 | 0.008 | 0.000 | 0.129 | 0.008 | 1.075 | 2.158 |
|  | 17th St. W | NM | NM | NM | NM | NM | NM | NM | NM | NM | NM | NM | NM | NM | NM |
|  | 23rd St. E | 0.000 | 4.945 | 0.069 | 0.000 | 0.137 | 0.072 | 0.000 | 0.043 | 0.034 | 0.922 | 0.000 | 0.069 | 0.060 | 6.351 |
|  | Silverwood Dog Park | 0.014 | 0.131 | 0.000 | 0.203 | 0.117 | 0.000 | 0.003 | 0.003 | 0.003 | 0.000 | 0.106 | 0.006 | 0.003 | 0.588 |
| August 22/19 | SCB E | 0.000 | 0.086 | 0.002 | 0.002 | 0.040 | 0.008 | 0.000 | 0.000 | 0.002 | 0.000 | 0.044 | 0.002 | 0.002 | 0.188 |
|  | SCB W | 0.116 | 0.205 | 0.000 | 0.000 | 0.000 | 0.011 | 0.015 | 0.011 | 0.033 | 0.037 | 0.049 | 0.018 | 0.009 | 0.505 |
|  | MacPherson Ave. | 0.000 | 0.148 | 0.000 | 0.008 | 0.047 | 0.000 | 0.006 | 0.002 | 0.002 | 0.000 | 0.062 | 0.004 | 0.004 | 0.284 |
|  | 14th St. E | 0.000 | 0.038 | 0.002 | 0.002 | 0.024 | 0.012 | 0.000 | 0.002 | 0.006 | 0.000 | 0.026 | 0.002 | 0.004 | 0.119 |
|  | 17th St. W | 0.130 | 0.070 | 0.168 | 0.174 | 0.083 | 0.108 | 0.137 | 0.119 | 0.094 | 0.009 | 0.021 | 0.137 | 0.136 | 1.385 |
|  | 23rd St. E | 0.078 | 0.055 | 0.027 | 0.026 | 0.027 | 0.041 | 0.009 | 0.010 | 0.039 | 0.033 | 0.026 | 0.020 | 0.012 | 0.402 |
|  | Silverwood Dog Park | 0.000 | 0.000 | 0.051 | 0.051 | 0.046 | 0.032 | 0.031 | 0.022 | 0.046 | 0.023 | 0.036 | 0.037 | 0.017 | 0.391 |
|  | CCME Guideline |  | 3 |  |  | 0.015 | 0.018 |  |  | 0.025 | 0.400 | 0.0012 |  |  |  |

**Table S9.** Theoretical seasonal loading estimates for various physicochemical parameters of interest. Estimates in this table are based on theoretical SMC values given in Table S3. Rainfall depths used to estimate seasonal catchment runoff volumes are included in Table A4. Estimates using measured seasonal mean concentrations are included in Table A10.

| **Catchment** | **Land Use** | **CR** | **% of area** | **Land Use Area, A (km^2^)** | **A * CR (km^2^)** | **Seasonal Runoff Volume (m^3^)** | **Seasonal Loading Estimate (kg)** | | | | | | | |
| --- | --- | --- | --- | --- | --- | --- | --- | --- | --- | --- | --- | --- | --- | --- |
|  |  |  |  |  |  |  | **TSS** | **COD** | **Cu** | **Cr** | **Ni** | **Pb** | **Zn** | **PAHs** |
| Wanuskewin Rd. | R | 0.95 | 4 | 1.02 | 0.97 | 131 | 35474 | 15708 | 7.20 | 2.62 | 3.80 | 14.92 | 31.02 | 0.10 |
|  | HW | 0.95 | 4 | 1.02 | 0.97 | 131 | 37700 | 15316 | 8.38 | 1.31 | 1.96 | 21.73 | 42.80 | 0.18 |
|  | IN | 0.6 | 37 | 9.43 | 5.66 | 765 | 148360 | 69592 | 64.24 | 10.71 | 18.35 | 111 | 199 | 0.46 |
|  | GR | 0.1 | 55 | 14.02 | 1.40 | 189 | 15915 | 7200 | 3.60 | 1.33 | 2.84 | 6.63 | 21.98 | 0.00 |
| **SUM** |  |  |  | **25** | **9.00** | 1216 | **237449** | **107815** | **83.42** | **15.96** | **26.96** | **154** | **295** | **0.75** |
| Circle Dr. S. Bridge | SR | 0.3 | 35 | 8.61 | 2.58 | 353 | 35314 | 20129 | 11.30 | 1.73 | 5.83 | 21.19 | 50.85 | 0.14 |
| (SCB W) | MR | 0.6 | 10 | 2.46 | 1.48 | 202 | 23812 | 13722 | 7.97 | 2.62 | 4.24 | 14.73 | 50.85 | 0.12 |
|  | R | 0.95 | 9 | 2.21 | 2.10 | 288 | 77929 | 34507 | 15.82 | 5.75 | 8.34 | 32.78 | 68.15 | 0.23 |
|  | HW | 0.95 | 7 | 1.72 | 1.64 | 224 | 64413 | 26168 | 14.31 | 2.24 | 3.35 | 37.13 | 73.14 | 0.31 |
|  | CM | 0.6 | 7 | 1.72 | 1.03 | 141 | 27404 | 12854 | 11.87 | 1.98 | 3.39 | 20.48 | 36.73 | 0.08 |
|  | IN | 0.6 | 15 | 3.69 | 2.21 | 303 | 58723 | 27545 | 25.43 | 4.24 | 7.26 | 43.89 | 78.70 | 0.18 |
|  | GR | 0.1 | 17 | 4.18 | 0.42 | 57 | 4803 | 2173 | 1.09 | 0.40 | 0.86 | 2.00 | 6.63 | 0.00 |
| **SUM** |  |  |  | **25** | **11.47** | 1567 | **292397** | **137098** | **87.78** | **18.96** | **33.27** | **172** | **365** | **1.07** |
| Light & Power | SR | 0.3 | 25 | 2.40 | 0.72 | 118 | 11811 | 6732 | 3.78 | 0.58 | 1.95 | 7.09 | 17.01 | 0.05 |
| (SCB E) | MR | 0.6 | 5 | 0.48 | 0.29 | 47 | 5575 | 3213 | 1.87 | 0.61 | 0.99 | 3.45 | 11.91 | 0.03 |
|  | R | 0.95 | 8 | 0.77 | 0.73 | 120 | 32434 | 14362 | 6.58 | 2.39 | 3.47 | 13.64 | 28.37 | 0.10 |
|  | HW | 0.95 | 8 | 0.77 | 0.73 | 120 | 34469 | 14003 | 7.66 | 1.20 | 1.80 | 19.87 | 39.14 | 0.17 |
|  | CM | 0.6 | 9 | 0.86 | 0.52 | 85 | 16497 | 7738 | 7.14 | 1.19 | 2.04 | 12.33 | 22.11 | 0.05 |
|  | IN | 0.6 | 25 | 2.40 | 1.44 | 236 | 45826 | 21496 | 19.84 | 3.31 | 5.67 | 34.25 | 61.42 | 0.14 |
|  | GR | 0.1 | 20 | 1.92 | 0.19 | 31 | 2646 | 1197 | 0.60 | 0.22 | 0.47 | 1.10 | 3.65 | 0.00 |
| **SUM** |  |  |  | **10** | **4.61** | 757 | **149258** | **68741** | **47.47** | **9.50** | **16.39** | **91.73** | **184** | **0.53** |
| 17th St. W. | SR | 0.3 | 39 | 3.61 | 1.08 | 178 | 17814 | 10154 | 5.70 | 0.87 | 2.94 | 10.69 | 25.65 | 0.07 |
|  | MR | 0.6 | 16 | 1.48 | 0.89 | 146 | 17248 | 9939 | 5.77 | 1.90 | 3.07 | 10.67 | 36.83 | 0.09 |
|  | R | 0.95 | 8 | 0.74 | 0.70 | 116 | 31359 | 13886 | 6.36 | 2.31 | 3.36 | 13.19 | 27.42 | 0.09 |
|  | HW | 0.95 | 5 | 0.46 | 0.44 | 72 | 20829 | 8462 | 4.63 | 0.72 | 1.08 | 12.01 | 23.65 | 0.10 |
|  | CM | 0.6 | 14 | 1.30 | 0.78 | 128 | 24812 | 11639 | 10.74 | 1.79 | 3.07 | 18.55 | 33.25 | 0.08 |
|  | IN | 0.6 | 5 | 0.46 | 0.28 | 46 | 8861 | 4157 | 3.84 | 0.64 | 1.10 | 6.62 | 11.88 | 0.03 |
|  | GR | 0.1 | 5 | 0.46 | 0.05 | 8 | 639 | 289 | 0.14 | 0.05 | 0.11 | 0.27 | 0.88 | 0.00 |
|  | AG | 0.05 | 5 | 0.46 | 0.02 | 4 | 320 | 145 | 0.07 | 0.03 | 0.06 | 0.13 | 0.44 | 0.00 |
| **SUM** |  |  |  | **9** | **4.24** | 697 | **121884** | **58671** | **37.26** | **8.32** | **14.79** | **72.12** | **160** | **0.46** |
| 14th St. E. | SR | 0.3 | 54 | 1.72 | 0.52 | 85 | 8464 | 4825 | 2.71 | 0.41 | 1.40 | 5.08 | 12.19 | 0.03 |
|  | MR | 0.6 | 12 | 0.38 | 0.23 | 38 | 4439 | 2558 | 1.49 | 0.49 | 0.79 | 2.75 | 9.48 | 0.02 |
|  | R | 0.95 | 9 | 0.29 | 0.27 | 45 | 12106 | 5361 | 2.46 | 0.89 | 1.30 | 5.09 | 10.59 | 0.04 |
|  | HW | 0.95 | 4 | 0.13 | 0.12 | 20 | 5718 | 2323 | 1.27 | 0.20 | 0.30 | 3.30 | 6.49 | 0.03 |
|  | CM | 0.6 | 10 | 0.32 | 0.19 | 31 | 6082 | 2853 | 2.63 | 0.44 | 0.75 | 4.55 | 8.15 | 0.02 |
|  | GR | 0.1 | 8 | 0.25 | 0.03 | 4 | 351 | 159 | 0.08 | 0.03 | 0.06 | 0.15 | 0.48 | 0.00 |
|  | AG | 0.05 | 3 | 0.10 | 0.00 | 1 | 66 | 30 | 0.01 | 0.01 | 0.01 | 0.03 | 0.09 | 0.00 |
| **SUM** |  |  |  | **3** | **1.36** | 223 | **37225** | **18107** | **10.65** | **2.47** | **4.61** | **20.93** | **47.47** | **0.14** |
| MacPherson Ave. | SR | 0.3 | 54 | 0.79 | 0.24 | 39 | 3913 | 2230 | 1.25 | 0.19 | 0.65 | 2.35 | 5.63 | 0.02 |
|  | MR | 0.6 | 12 | 0.18 | 0.11 | 17 | 2052 | 1182 | 0.69 | 0.23 | 0.37 | 1.27 | 4.38 | 0.01 |
|  | R | 0.95 | 9 | 0.13 | 0.13 | 21 | 5596 | 2478 | 1.14 | 0.41 | 0.60 | 2.35 | 4.89 | 0.02 |
|  | HW | 0.95 | 4 | 0.06 | 0.06 | 9 | 2643 | 1074 | 0.59 | 0.09 | 0.14 | 1.52 | 3.00 | 0.01 |
|  | CM | 0.6 | 10 | 0.15 | 0.09 | 14 | 2811 | 1319 | 1.22 | 0.20 | 0.35 | 2.10 | 3.77 | 0.01 |
|  | GR | 0.1 | 8 | 0.12 | 0.01 | 2 | 162 | 73 | 0.04 | 0.01 | 0.03 | 0.07 | 0.22 | 0.00 |
|  | AG | 0.05 | 3 | 0.04 | 0.00 | 0 | 30 | 14 | 0.01 | 0.00 | 0.01 | 0.01 | 0.04 | 0.00 |
| **SUM** |  |  |  | **1** | **0.63** | 103 | **17208** | **8370** | **4.92** | **1.14** | **2.13** | **9.68** | **21.95** | **0.06** |
| 23rd St. W. | SR | 0.3 | 20 | 0.55 | 0.16 | 27 | 2695 | 1536 | 0.86 | 0.13 | 0.44 | 1.62 | 3.88 | 0.01 |
|  | MR | 0.6 | 6 | 0.16 | 0.10 | 16 | 1908 | 1100 | 0.64 | 0.21 | 0.34 | 1.18 | 4.07 | 0.01 |
|  | R | 0.95 | 10 | 0.27 | 0.26 | 43 | 11564 | 5120 | 2.35 | 0.85 | 1.24 | 4.86 | 10.11 | 0.03 |
|  | HW | 0.95 | 6 | 0.16 | 0.16 | 26 | 7373 | 2995 | 1.64 | 0.26 | 0.38 | 4.25 | 8.37 | 0.04 |
|  | CM | 0.6 | 29 | 0.79 | 0.48 | 78 | 15162 | 7112 | 6.56 | 1.09 | 1.88 | 11.33 | 20.32 | 0.05 |
|  | IN | 0.6 | 25 | 0.68 | 0.41 | 67 | 13070 | 6131 | 5.66 | 0.94 | 1.62 | 9.77 | 17.52 | 0.04 |
|  | GR | 0.1 | 4 | 0.11 | 0.01 | 2 | 151 | 68 | 0.03 | 0.01 | 0.03 | 0.06 | 0.21 | 0.00 |
| **SUM** |  |  |  | **3** | **1.57** | 259 | **51923** | **24063** | **17.74** | **3.50** | **5.93** | **33.08** | **64.49** | **0.18** |
| **TOTAL SUM** |  |  |  |  |  |  | **907345** | **422866** | **289.2** | **59.9** | **104.1** | **553.9** | **1137.2** | **3.19** |

#### Table S10. Correlations between runoff volume from land use area across each catchment (calculated from surface area; see Methods) and contaminant loading. Loading is based upon averages of measured 2019 SW concentrations; see Table S5.

| **LAND USE** | **Total surface area** | **Contaminant loading (kg)** | | | | | | | |
| --- | --- | --- | --- | --- | --- | --- | --- | --- | --- |
| **ROAD** | **R+HW area (km^2^)** | **TDS** | **TSS** | **COD** | **Cu** | **Ni** | **Pb** | **Zn** | **PAHs** |
| SCB E | 1.53 | 68280 | 12058 | 39256 | 17.1 | 0.440 | 1.12 | 45.3 | 0.187 |
| SCB W | 3.94 | 145824 | 114385 | 144674 | 22.3 | 4.42 | 0.869 | 122 | 1.01 |
| MacPherson Ave. | 0.235 | 8508 | 6838 | 17543 | 1.23 | 0.151 | 0.039 | 6.38 | 0.014 |
| 14th St. E. | 0.413 | 18406 | 15357 | 31787 | 2.19 | 0.304 | 0.122 | 12.3 | 0.050 |
| 17th St. W. | 1.30 | 19475 | 35885 | 42311 | 3.66 | 0.934 | 0.140 | 14.5 | 0.124 |
| 23rd St. W. | 0.437 | 53639 | 89084 | 176734 | 7.20 | 1.38 | 0.403 | 31.7 | 0.115 |
| Silverwood Dog Park | 2.04 | 74680 | 56910 | 121706 | 10.5 | 1.75 | 0.333 | 117 | 0.165 |
| **Correlation (R)** |  | **0.840** | **0.425** | **0.188** | **0.748** | **0.808** | **0.374** | **0.754** | **0.847** |
| **INDUSTRIAL** | **IN area (km^2^)** | **TDS** | **TSS** | **COD** | **Cu** | **Ni** | **Pb** | **Zn** | **PAHs** |
| SCB E | 2.40 | 67381 | 11899 | 38740 | 16.83 | 0.434 | 1.11 | 44.7 | 0.185 |
| SCB W | 3.69 | 86343 | 67728 | 85662 | 13.23 | 2.62 | 0.515 | 72.5 | 0.599 |
| MacPherson Ave. | 0 |  |  |  |  |  |  |  |  |
| 14th St. E. | 0 |  |  |  |  |  |  |  |  |
| 17th St. W. | 0.464 | 13030 | 21640 | 42931 | 1.75 | 0.336 | 0.098 | 7.71 | 0.028 |
| 23rd St. W. | 0.683 | 19218 | 35413 | 41755 | 3.61 | 0.922 | 0.138 | 14.3 | 0.123 |
| Silverwood Dog Park | 9.44 | 218143 | 166236 | 355510 | 30.67 | 5.12 | 0.97 | 343 | 0.482 |
| **Correlation (R)** |  | **0.997** | **0.902** | **0.927** | **0.900** | **0.915** | **0.425** | **0.966** | **0.518** |
| **COMMERCIAL** | **CM area (km^2^)** | **TDS** | **TSS** | **COD** | **Cu** | **Ni** | **Pb** | **Zn** | **PAHs** |
| SCB E | 0.862 | 24257 | 4284 | 13946 | 6.06 | 0.16 | 0.40 | 16.1 | 0.07 |
| SCB W | 1.72 | 40294 | 31606 | 39976 | 6.17 | 1.22 | 0.24 | 33.8 | 0.28 |
| MacPherson Ave. | 0.132 | 4134 | 3322 | 8523 | 0.60 | 0.07 | 0.02 | 3.10 | 0.01 |
| 14th St. E. | 0.350 | 8942 | 7461 | 15443 | 1.06 | 0.15 | 0.06 | 5.96 | 0.02 |
| 17th St. W. | 1.30 | 36483 | 60592 | 120208 | 4.90 | 0.94 | 0.27 | 21.6 | 0.08 |
| 23rd St. W. | 0.792 | 22293 | 41079 | 48435 | 4.19 | 1.07 | 0.16 | 16.6 | 0.14 |
| Silverwood Dog Park | 0 |  |  |  |  |  |  |  |  |
| **Correlation (R)** |  | **0.973** | **0.419** | **0.322** | **0.753** | **0.661** | **0.432** | **0.978** | **0.716** |
| **SINGLE RESIDENTIAL** | **SR area (km^2^)** | **TDS** | **TSS** | **COD** | **Cu** | **Ni** | **Pb** | **Zn** | **PAHs** |
| SCB E | 2.87 | 33691 | 5950 | 19370 | 8.41 | 0.217 | 0.553 | 22.4 | 0.092 |
| SCB W | 11.1 | 100734 | 79016 | 99939 | 15.4 | 3.05 | 0.600 | 84.6 | 0.698 |
| MacPherson Ave. | 0.441 | 11161 | 8970 | 23011 | 1.61 | 0.198 | 0.051 | 8.36 | 0.018 |
| 14th St. E. | 2.10 | 24144 | 20145 | 41696 | 2.88 | 0.40 | 0.160 | 16.1 | 0.065 |
| 17th St. W. | 5.28 | 50815 | 84395 | 167433 | 6.82 | 1.31 | 0.382 | 30.1 | 0.109 |
| 23rd St. W. | 0.710 | 7687 | 14165 | 16702 | 1.44 | 0.369 | 0.055 | 5.72 | 0.049 |
| Silverwood Dog Park | 0 |  |  |  |  |  |  |  |  |
| **Correlation (R)** |  | **0.995** | **0.684** | **0.413** | **0.895** | **0.944** | **0.632** | **0.973** | **0.888** |
| **MULTI-RESIDENTIAL** | **MR area (km^2^)** | **TDS** | **TSS** | **COD** | **Cu** | **Ni** | **Pb** | **Zn** | **PAHs** |
| SCB E | 2.87 | 13476 | 2380 | 7748 | 3.37 | 0.087 | 0.221 | 8.95 | 0.037 |
| SCB W | 11.1 | 57562 | 45152 | 57108 | 8.82 | 1.74 | 0.343 | 48.3 | 0.399 |
| MacPherson Ave. | 0.441 | 4960 | 3987 | 10227 | 0.716 | 0.088 | 0.023 | 3.72 | 0.008 |
| 14th St. E. | 2.10 | 10731 | 8953 | 18532 | 1.28 | 0.177 | 0.071 | 7.16 | 0.029 |
| 17th St. W. | 5.28 | 41695 | 69248 | 137381 | 5.59 | 1.07 | 0.313 | 24.7 | 0.090 |
| 23rd St. W. | 0.710 | 4612 | 8499 | 10021 | 0.867 | 0.221 | 0.033 | 3.43 | 0.029 |
| Silverwood Dog Park | 0 |  |  |  |  |  |  |  |  |
| **Correlation (R)** |  | **0.932** | **0.478** | **0.268** | **0.958** | **0.907** | **0.765** | **0.986** | **0.920** |
| **GREEN + AG** | **GR+AG area (km^2^)** | **TDS** | **TSS** | **COD** | **Cu** | **Ni** | **Pb** | **Zn** | **PAHs** |
| SCB E | 1.92 | 8984 | 1587 | 5165 | 2.24 | 0.058 | 0.15 | 5.96 | 0.025 |
| SCB W | 4.18 | 16309 | 12793 | 16181 | 2.50 | 0.494 | 0.10 | 13.7 | 0.113 |
| MacPherson Ave. | 0.162 | 654 | 526 | 1349 | 0.095 | 0.012 | 0.00 | 0.490 | 0.001 |
| 14th St. E. | 0.350 | 1416 | 1181 | 2445 | 0.169 | 0.023 | 0.01 | 0.944 | 0.004 |
| 17th St. W. | 0.927 | 654 | 526 | 1349 | 0.095 | 0.012 | 0.00 | 0.490 | 0.001 |
| 23rd St. W. | 0.109 | 512 | 944 | 1113 | 0.096 | 0.025 | 0.00 | 0.381 | 0.003 |
| Silverwood Dog Park | 14.02 | 54044 | 41185 | 88077 | 7.60 | 1.27 | 0.24 | 85.0 | 0.119 |
| **Correlation (R)** |  | **0.013** | **0.010** | **0.048** | **0.009** | **0.001** | **0.001** | **0.054** | **0.145** |

#####
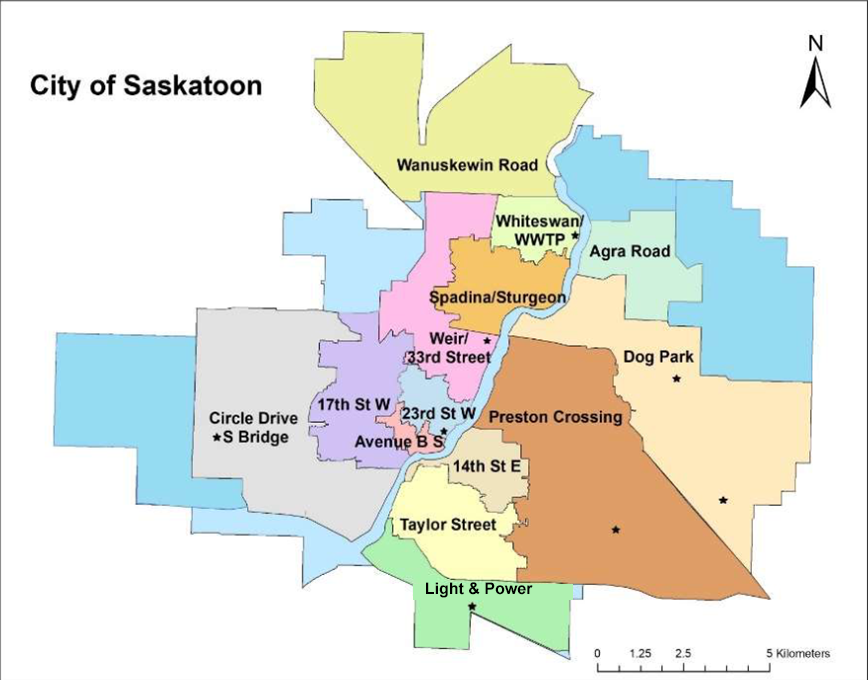


##### **Figure S1.** Stormwater catchments delineated within the CoS following Al Masum et al. (2021). Catchments included in this study are Light & Power (SCB E), Circle Drive S Bridge (SCB W), 14^th^ St. E (including both the MacPherson Ave. and 14^th^ St. E catchments), 17^th^ St. W, 23^rd^ St. W, and Wanuskewin Road (Silverwood Dog Park). Stormwater sampling occurred over summer 2019; the data is intended to complement a parallel 2018 study examining summer SW quality in the Taylor Street, Avenue B S, Preston Crossing, Dog Park (Preston), Spadina/Sturgeon, and Whiteswan/WWTP catchments.

**Figure S2.** Land use breakdown of study catchments. The 14^th^ St. E catchment above drains to both the MacPherson Ave. and 14^th^ St E outfalls (though not delineated on the land-use map, areas for each subcatchment are provided by the CoS). Adapated from Al Masum *et al. (2021)*. Refer to Table S1 for land use classification acronyms. Refer to Table S2 for total and land-use surface area in km^2^.


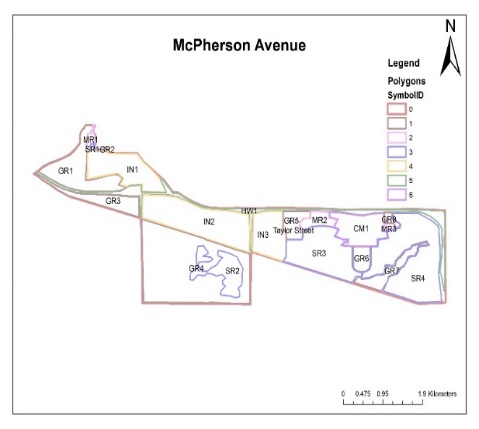

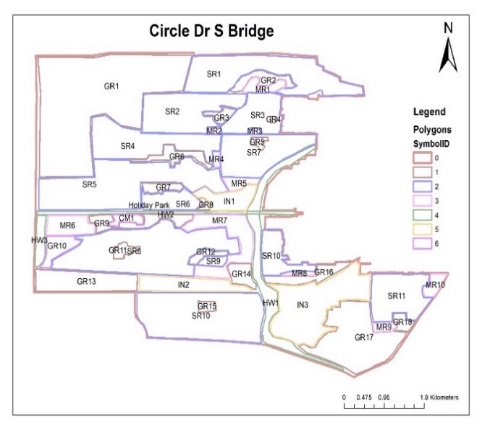

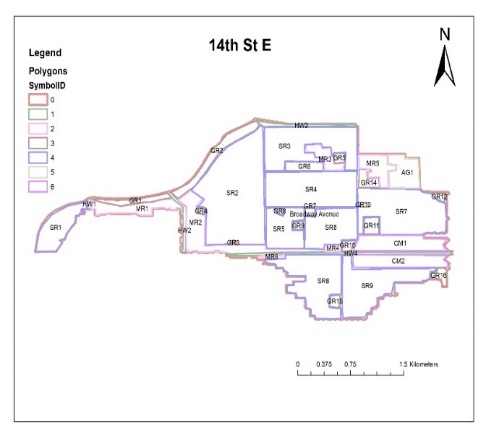

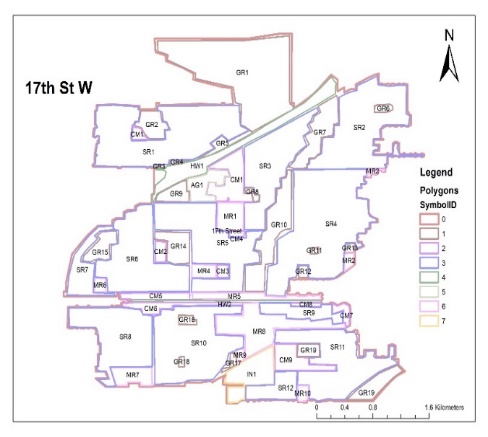

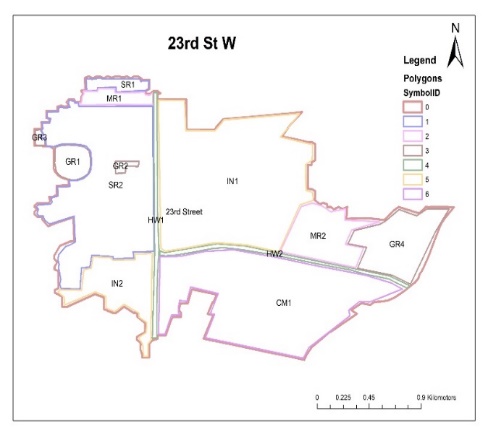

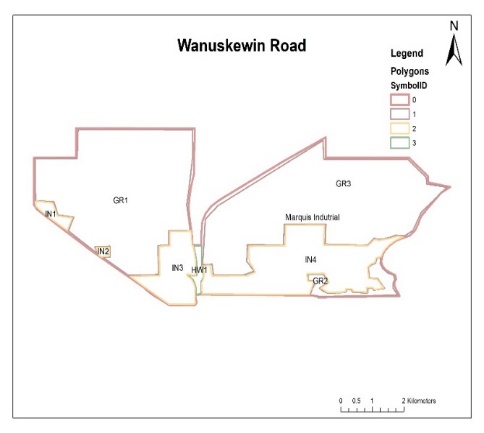

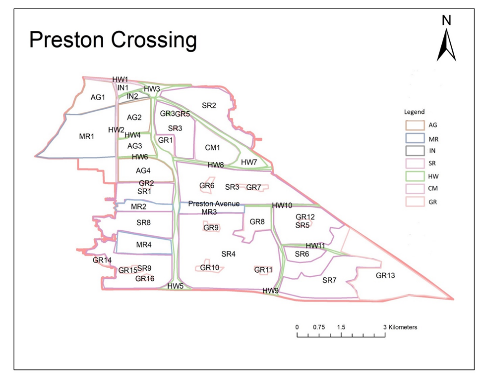

Supplement: Supplementary file 1 — Additional file 1: Table S1. Limits of detection (LOD) and quantification (LOQ) for polyaromatic hydrocarbons. Table S2. Land use classifications used to calculate runoff volumes in this study based on Järveläinen et al. [39]. The runoff coefficients (CR) considered in the current study follow City of Saskatoon (CoS) stormwater management guidelines. Table S3. Average flow-weighted site mean concentration (SMC) values for different land use classes, from Melanen (1981), Mitchell (2005), Nordeidet et al. (2004) and Järveläinen et al. [39] as adapted by Al Masum et al. [3]. The SMC is the geometric mean of the event mean concentration for each storm event, which is the concentration of pollutants as a function of the runoff volume discharging in the river (flow-weighted). The value is used to estimate overall SW contaminant loading over a given urban area. Values in parentheses are standard deviations (SD). Table S4. Overview of analyzed stormwater quality parameters for the 2019 sampling season grouped by site. Catchments outlined in Additional file 1: Figure S1. Values are average (standard deviation, SD). Quality parameter abbreviations are as follows: total dissolved solids (TDS), electrical conductivity (EC), dissolved organic carbon (DOC), chemical oxygen demand (COD), and total suspended solids (TSS). Table S5. Overview of stormwater quality parameters for the 2019 sampling season for each individual event and outfall. Catchments outlined in Additional file 1: Figure S1. Table S6. Chloride and sulphate analysis for select 2019 stormwater samples. Table S7. Metals (µg/L) detected in 2019 stormwater samples. Table S8. PAHs (ng/L) detected in 2019 stormwater samples. NM not measured. Table S9. Theoretical seasonal loading estimates for various physicochemical parameters of interest. Estimates in this table are based on theoretical SMC values given in Additional file 1: Table S3. Rainfall depths used to estimate seasonal catchment runoff volumes are included in Add [file 12302_2022_619_MOESM1_ESM.docx]
